# Design, Synthesis, In Vitro, and Initial In Vivo Evaluation of Heterobivalent Peptidic Ligands Targeting Both NPY(Y_1_)- and GRP-Receptors—An Improvement for Breast Cancer Imaging?

**DOI:** 10.3390/ph11030065

**Published:** 2018-07-04

**Authors:** Alicia Vall-Sagarra, Shanna Litau, Clemens Decristoforo, Björn Wängler, Ralf Schirrmacher, Gert Fricker, Carmen Wängler

**Affiliations:** 1 Biomedical Chemistry, Department of Clinical Radiology and Nuclear Medicine, Medical Faculty Mannheim of Heidelberg University, Theodor-Kutzer-Ufer 1-3, 68167 Mannheim, Germany; Alicia.Vall-Sagarra@medma.uni-heidelberg.de (A.V.-S.); Shanna.Litau@medma.uni-heidelberg.de (S.L.); 2 Molecular Imaging and Radiochemistry, Department of Clinical Radiology and Nuclear Medicine, Medical Faculty Mannheim of Heidelberg University, Theodor-Kutzer-Ufer 1-3, 68167 Mannheim, Germany; Bjoern.Waengler@medma.uni-heidelberg.de; 3 Department of Nuclear Medicine, University Hospital Innsbruck, Medical University Innsbruck, Anichstrasse 35, 6020 Innsbruck, Austria; Clemens.Decristoforo@tirol-kliniken.at; 4 Department of Oncology, Division Oncological Imaging, University of Alberta, 11560 University Avenue, Edmonton, AB T6G 1Z2, Canada; schirrma@ualberta.ca; 5 Institute of Pharmacy and Molecular Biotechnology, University of Heidelberg, Im Neuenheimer Feld 329, 69120 Heidelberg, Germany; gert.fricker@uni-hd.de

**Keywords:** breast cancer, ^68^Ga, GRPR, NPY(Y_1_)R, peptide heterodimers, PET/CT imaging

## Abstract

Heterobivalent peptidic ligands (HBPLs), designed to address two different receptors independently, are highly promising tumor imaging agents. For example, breast cancer has been shown to concomitantly and complementarily overexpress the neuropeptide Y receptor subtype 1 (NPY(Y_1_)R) as well as the gastrin-releasing peptide receptor (GRPR). Thus, radiolabeled HBPLs being able to bind these two receptors should exhibit an improved tumor targeting efficiency compared to monospecific ligands. We developed here such bispecific HBPLs and radiolabeled them with ^68^Ga, achieving high radiochemical yields, purities, and molar activities. We evaluated the HBPLs and their monospecific reference peptides in vitro regarding stability and uptake into different breast cancer cell lines and found that the ^68^Ga-HBPLs were efficiently taken up via the GRPR. We also performed in vivo PET/CT imaging and ex vivo biodistribution studies in T-47D tumor-bearing mice for the most promising ^68^Ga-HBPL and compared the results to those obtained for its scrambled analogs. The tumors could easily be visualized by the newly developed ^68^Ga-HBPL and considerably higher tumor uptakes and tumor-to-background ratios were obtained compared to the scrambled analogs in and ex vivo. These results demonstrate the general feasibility of the approach to use bispecific radioligands for in vivo imaging of breast cancer.

## 1. Introduction

Radiolabeled peptides, being able to specifically bind certain receptors overexpressed on many malignancies, have become standard radiotracers for tumor-specific imaging by positron emission tomography (PET) in a clinical routine. However, these radiolabeled peptides are able to address only one target receptor type, being thus only able to visualize tumors expressing this particular receptor. As different tumor lesions can however overexpress different receptor types and receptor expression can change upon metastasis and disease progression, a monovalent peptidic radioligand is often not able to visualize all tumor cells with the same efficiency and some lesions might be completely missed by the applied radiopeptide.

Radiolabeled heterobivalent peptide ligands (HBPLs) on the other hand—by their ability to specifically target more than one receptor type—have been proposed to be better-suited agents for tumor imaging as they enable the visualization of the tumor by different receptor types potentially overexpressed by the target cells [1,2].

HBPLs furthermore can have favorable effects for in vivo tumor imaging compared to monovalent ligands such as improved in vivo biodistribution and enhanced avidity caused by simultaneous binding if both receptors are present on the tumor cell surface. In this case, also a higher probability of rebinding is achieved for a heterobivalent binder in case of dissociation from the receptor compared to a monovalent peptide due to “forced proximity” of the second potential binder to the second target receptor.

**Figure 1:**
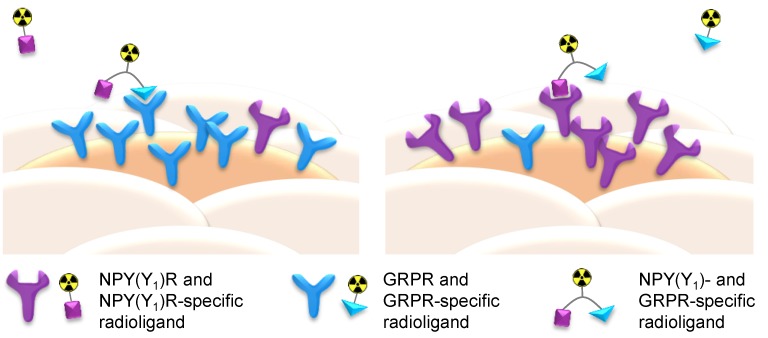
Schematic depiction of the functional principle of radiolabeled NPY(Y_1_)R- and gastrin-releasing peptide receptor (GRPR)-binding heterobivalent peptidic ligands (HBPLs) for tumor imaging: Monomeric peptide radiotracers can only bind to one receptor type and thus miss tumor tissues or lesions that do not express the respective receptor due to tumor heterogeneity or disease progression whereas the use of radiolabeled HBPLs, which can bind to more than one target receptor type, results in a higher probability of target visualization.

Furthermore, by being able to target different receptor types on the tumor cell surface, an overall higher number of receptors can be addressed by a heterobivalent ligand, increasing the probability of binding and thus tumor visualization. Thus, HBPLs are also of special interest not only for the imaging of such tumors that express both receptor types concomitantly, but also for tumors exhibiting a heterogeneous target expression with varying receptor densities between different individuals or lesions and for differential target expression during disease progression. A non-uniform distribution of target receptors between lesions results—if using monomeric peptidic radioligands—in the visualization of only some of the lesions whereas others are not depicted. Applying a HBPL for imaging, such lesions can nevertheless be addressed as long as one of the target receptors is present (Figure 1).

Recently, a radiolabeled HBPL, being able to address the gastrin-releasing peptide receptor as well as integrin α_v_β_3_ was successfully translated into the clinics for imaging of prostate cancer with PET/CT, showing a much higher tumor visualization sensitivity compared to the respective GRPR-targeting peptide monomer. This proves the clinical relevance of the heterobivalent peptide targeting concept [3].

To be able to develop a HBPL being able to more efficiently target tumor tissues than the respective peptide monomers, it is necessary to know the receptor expression profile on the target tumor type. Regarding this point, some excellent systematical work has been carried out, determining the presence of certain receptor types and their densities on different human malignancies [2,4,5,6].

Human breast cancer, for example, overexpresses in about 75% of all cases the gastrin-releasing peptide receptor (GRPR) [7] and in 66–85% the neuropeptide Y receptor subtype 1 (NPY(Y_1_)R) [8]. Both receptors are expressed to an insignificant amount on healthy breast tissue, thus rendering both receptor types well-suited target structures for sensitive and specific breast cancer imaging. Furthermore, Reubi and co-workers could show on 68 human breast cancer samples that 63/68 (93%) overexpressed one or both receptor types. Of these, 32 (51%) expressed both receptors concomitantly, whereas 18 (29%) expressed only the GRPR, and a further 13 (21%) expressed only the NPY(Y_1_)R [5]. Thus, the combination of two peptidic ligands being able to specifically bind the NPY(Y_1_)R and the GRPR to one radioligand should enable a considerably higher breast cancer visualization efficiency and sensitivity and thus give less false-negative results compared to the respective monovalent radioligands (Figure 1).

To obtain a highly potent bispecific HBPL, it is mandatory that both peptide parts of the construct are still able to bind to their respective target receptor type despite the considerable chemical modifications necessary for peptide heterodimerization. Thus, a suitable molecular design has to be found that enables the binding of both peptides to their respective target receptor.

So far, only one example of a heterobivalent NPY(Y_1_)R- and GRPR-targeting ligand has been described [9,10] and for this substance, no descriptions of radiolabeling with a PET isotope, in vitro cell uptake or in vivo imaging data are available. Thus, the general feasibility of the approach has not been demonstrated so far.

Thus, the aims of this study were to: (i) Develop a synthesis strategy yielding different HBPLs varying in molecular design and consisting of a NPY(Y_1_)- and a GRPR-affine peptide as well as a chelating agent (for radiolabeling with the positron-emitting radiometal nuclide ^68^Ga); (ii) Establish the ^68^Ga-radiolabeling and determine the log*_D_* and in vitro stability of the resulting ^68^Ga-HBPLs in human serum; (iii) Evaluate the uptake of the ^68^Ga-HBPLs into human breast cancer cell lines in vitro to determine if the substances can still interact with both target receptors and are taken up by the tumor cells; (iv) Determine if the particular molecular design used has a measurable influence on tumor cell uptake; and (v) Show the general feasibility of the approach by investigating the tumor uptake of the most potent HBPL in vivo by PET/CT imaging and ex vivo biodistribution in a proof-of-concept study and determine if the tumor uptake profits from the heterodimerization of the receptor-specific peptides.

## 2. Results and Discussion

### 2.1. Synthesis of GRPR- and NPY(Y_1_)R-Binding HBPLs ***22***–***26***, Scrambled HBPL Analogs ***24a***–***c***, Blocking Agents ***3*** and ***4*** as well as Monomeric Reference Peptides ***27*** and ***28***

**Figure 2:**
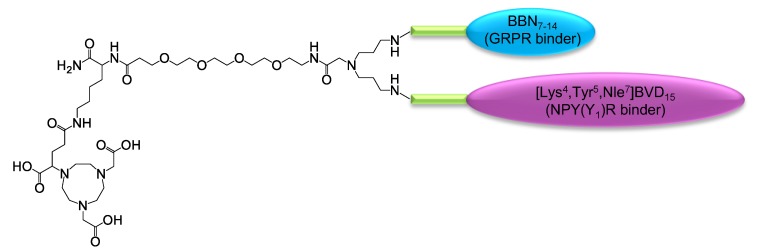
Schematic depiction of the general molecular design of the target HBPLs consisting of a chelating agent (NODA-GA), a short PEG_4_-linker between radiometal complex and peptides, the symmetrical branching unit, the linkers of different length and rigidity (green) and the GRPR- and NPY(Y_1_)R-binding peptides BBN_7–14_ (cyan) and [Lys^4^,Trp^5^,Nle^7^]BVD_15_ (magenta).

At first, a suitable synthesis strategy towards the GRPR- and NPY(Y_1_)R-binding HBPLs was developed. The target molecular design of the substances is depicted in Figure 2 and was based on the following considerations: (i) The structure was to be based on a symmetrically branched scaffold to obtain homogenous products and the scaffold should comprise the chelator NODA-GA ((1,4,7-triazacyclononane-4,7-diyl)diacetic acid-1-glutaric acid), which is able to stably and efficiently complex ^68^Ga [11]; (ii) The chelator should be spatially separated from the receptor-affine peptides by a short PEG linker to prevent an interference of the radiometal complex with receptor binding [12]; (iii) As we and others were able to show before for peptide di- and multimers, the distance between the peptides within the same molecule can have a significant influence on the achievable receptor interaction [13,14,15,16], thus different distances between both peptidic receptor ligands should be investigated for the target HBPLs by introducing linkers of different length; (iv) Furthermore, as it was proposed that also the rigidity of the molecules might influence cellular uptakes by a conformational stabilization of the spatial orientation of the receptor ligands, the used linker structures did not only differ in length, but also in rigidity.

#### 2.1.1. Synthesis of the Peptide Monomers **1**−**6**

Aiming at the synthesis of GRPR-and NPY(Y_1_)R-binding HBPLs and their following in vitro and in vivo evaluation in human breast cancer tumor cells, we first synthesized the respective peptide monomers for subsequent heterodimerization on symmetrically branched scaffolds. As GRPR-affine peptide monomer, we chose the receptor agonist PESIN (PEG_4_-BBN_7–14_), which exhibits a favorably high stability, significant tumor uptake, as well as high tumor to background ratios in vivo [17,18], and can be modified at its *N*-terminal end without considerably changing its receptor binding affinity [13]. As NPY(Y_1_)R-affine peptide monomer, we chose [Lys^4^,Trp^5^,Nle^7^]BVD_15_ as this peptide was shown to exhibit good affinities to the NPY(Y_1_)R even when further modified in position four [9,19,20].

The peptides were to be conjugated to the symmetrically branched scaffolds by a click chemistry approach to be able to obtain the desired rather complex target HBPLs efficiently. Furthermore, the coupling products have to be stable under physiological conditions. Different click chemistry reactions fulfill these requirements; of these, we chose the oxime formation between aminooxy functionalities and aldehydes.

**Figure 3:**
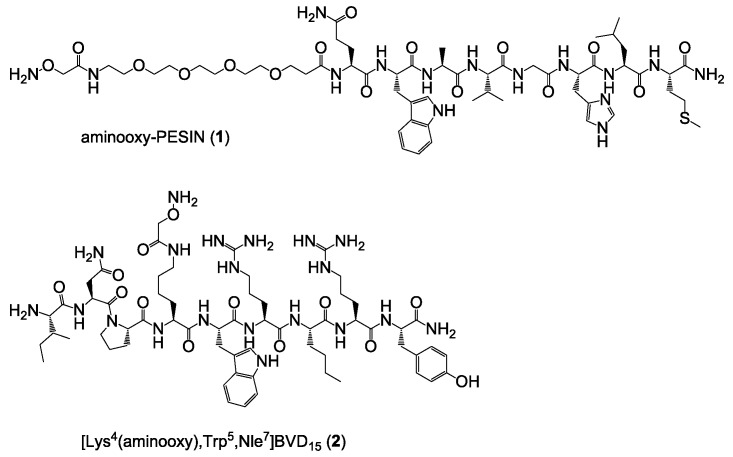
Depiction of the chemical structures of aminooxy-PESIN (**1**) and [Lys^4^(aminooxy),Trp^5^,Nle^7^]BVD_15_ (**2**) used for the assembly of the HBPLs.

Both peptides were synthesized by standard solid phase peptide synthesis (SPPS) methods [13,21] by successive conjugation of the respective *N*_α_-Fmoc-amino acids after HBTU activation to the respective rink amide resin and finally modified on resin with *bis*-Boc-aminooxy acetic acid, giving aminooxy-PESIN (**1**) and [Lys^4^(aminooxy),Trp^5^,Nle^7^]BVD_15_ (**2**) (Figure 3). In case of PESIN, the aminooxy functionality was introduced at the *N*-terminal end as the peptide can be modified in this position without considerable alterations in binding affinity. In case of [Lys^4^,Trp^5^,Nle^7^]BVD_15_, the aminooxy functionality was introduced in position 4 (*N*_ε_ amine of lysine) as modifications in this position interfere least with receptor binding.

**Figure 4:**
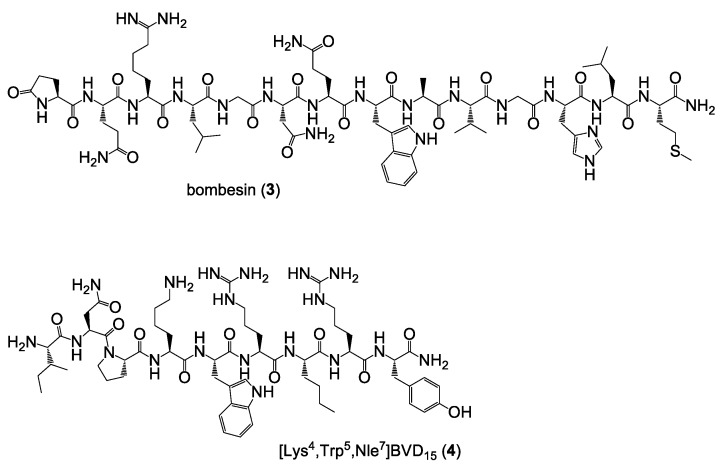
Depiction of the chemical structures of bombesin (**3**) and [Lys^4^,Trp^5^,Nle^7^]BVD_15_ (**4**) used as receptor blocking substances during the in vitro tumor cell uptake studies.

As the target HBPLs should be evaluated in vitro regarding their ability to be taken up by human breast cancer cell lines and the contribution of both parts of the HBPLs on tumor cell uptake should be assessed, we further synthesized the peptide monomers bombesin (**3**) and [Lys^4^,Trp^5^,Nle^7^]BVD_15_ (**4**) as blocking substances for the GRPR and the NPY(Y_1_)R during these experiments (Figure 4).

Regarding the in vivo evaluation of the HBPLs in tumor-bearing animals and the verification of the receptor specificity of the observed tumor uptakes and the contribution of both peptides of the HBPLs to overall tumor uptakes, two different approaches can be followed. The first one is to block the respective target receptor analogous to the in vitro assays by adding blocking substances **3** or **4** and the other one is to use scrambled HBPL analogs. To compare the in vivo tumor uptake of an HBPL to that of its scrambled analogs instead of performing blocking studies however eliminates possible difficulties that might arise from the low stability of the monomeric receptor ligands.

**Figure 5:**
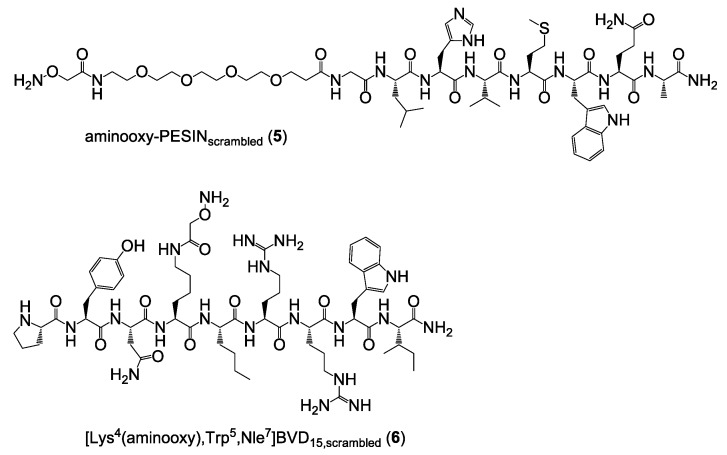
Depiction of the chemical structures of aminooxy-PESIN_scrambled_ (**5**) and [Lys^4^(aminooxy),Trp^5^,Nle^7^]BVD_15,scrambled_ (**6**) which were used to synthesize partly or fully scrambled HBPL analogs.

Thus, three different scrambled HBPL analogs were synthesized: PESIN combined with scrambled [Lys^4^(aminooxy),Trp^5^,Nle^7^]BVD_15_, scrambled PESIN combined with [Lys^4^(aminooxy),Trp^5^,Nle^7^]BVD_15_ and both peptides of the HBPL scrambled. For this purpose, the two scrambled aminooxy-modified peptide monomers aminooxy-PESIN_scrambled_ (**5**) and [Lys^4^(aminooxy),Trp^5^,Nle^7^]BVD_15,scrambled_ (**6**) (Figure 5) were synthesized and analogously to **1** and **2** used during the following HBPL syntheses. 

#### 2.1.2. Synthesis of the Heterobivalent Ligands **22**–**26**, **24a**–**c** and Monomeric Reference Peptides **27** and **28**

**Scheme 1:**
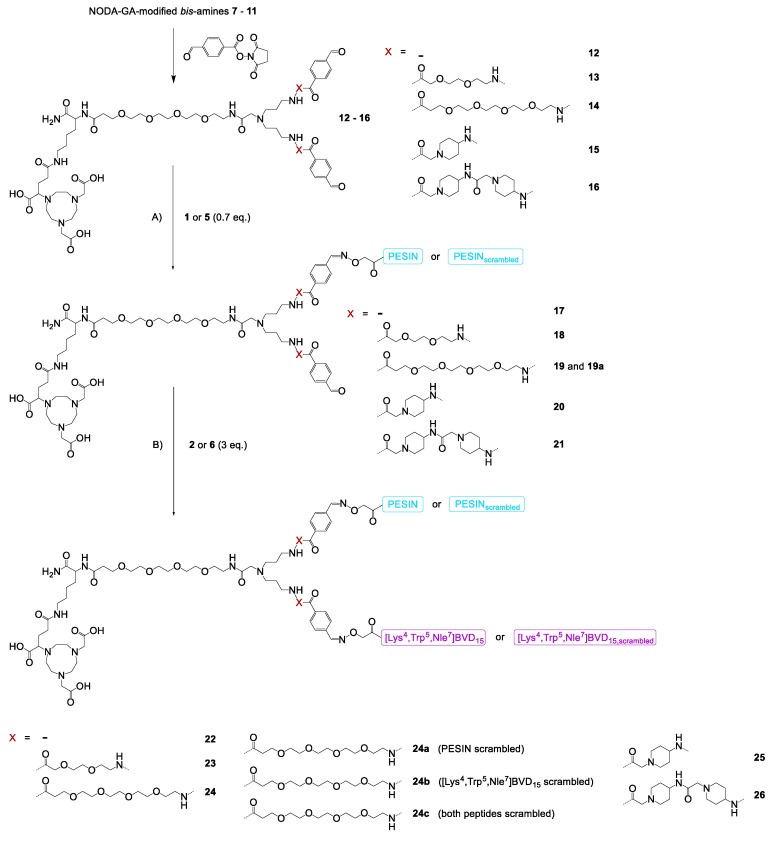
Schematic depiction of the syntheses of the GRPR- and NPY(Y_1_)R-affine HBPLs **22**−**26** and the scrambled analogs **24a**–**c**. Conditions: (**A**) **1**, H_2_O + 0.1% TFA, phosphate buffer, pH 4.0−4.6, RT, 5 min, yields: 47% for **17**, 51% for **18**, 43% for **19**, 44% for **19a**, 58% for **20**, 55% for **21**; (**B**) **2**, H_2_O + 0.1% TFA, phosphate buffer, pH 4.0−4.6, RT, 5 min, yields: 82% for **22**, 79% for **23**, 66% for **24**, 49% for **24a**, 63% for **24b**, 61% for **24c**, 75% for **25**, 73% for **26**.

The branched *bis*-amines **7**−**11** and *bis*-aldehydes **12**−**16** (Scheme 1) were synthesized following a published procedure [22] with minor modifications (see Supplementary Materials for detailed description). In the following, these NODA-GA-modified branched *bis*-aldehyde scaffolds **12**−**16** were efficiently reacted with aminooxy-PESIN (**1**) and aminooxy-PESIN_scrambled_ (**5**) to the monovalent intermediates **17**−**21** and **19a**. These were further reacted with [Lys^4^(aminooxy),Trp^5^,Nle^7^]BVD_15_ (**2**) and its scrambled analog **6** to the final heterobivalent peptidic target structures **22**−**26** and their partly or fully scrambled analogs **24a**–**c** (Scheme 1).

**1** as well as its scrambled analog **5** reacted efficiently within minutes with the branched *bis*-aldehydes **12**−**16**, giving the respective monovalent conjugation products **17**−**21** and **19a** in satisfactory yields of 47% to 58%. Higher yields could not be obtained as **1** and **5** had to be applied in a lower amount than the *bis*-aldehydes **12**−**16** to minimize the formation of the respective homobivalent PESIN-dimers, being the only observed side products in this reaction. These monovalent intermediates were in the following reacted with **2** or **6**, proceeding equally efficient than the first reaction step within minutes, giving the target HBPLs **22**−**26** as well as the scrambled analogs **24a**–**c** in good yields of 49% to 82%.

The HBPLs exhibited—depending on the linker structure used—distances between both peptidic receptor ligands of 46 (no additional linker units used), 64 (PEG_2_ linkers), 78 (PEG_4_ linkers), 60 (ACMP linkers), and 74 (two successive ACMP linkers) bond lengths.

**Figure 6:**
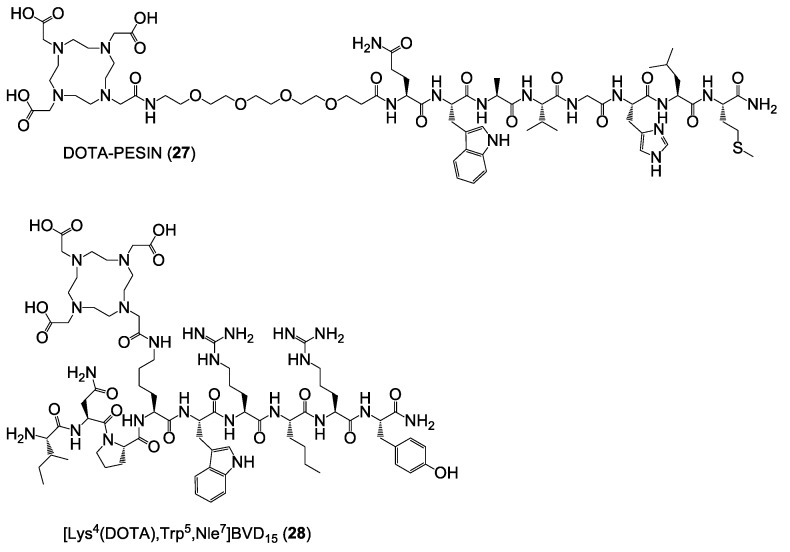
Depiction of the structures of DOTA-PESIN (**27**) and [Lys^4^(DOTA),Trp^5^,Nle^7^]BVD_15_ (**28**), serving as mono-specific reference substances for the HBPLs in the following in vitro tumor cell uptake studies.

Besides the target HBPLs and the scrambled analogs, also the monomeric reference compounds DOTA-PESIN (**27**) [18] and [Lys^4^(DOTA),Trp^5^,Nle^7^]BVD_15_ (**28**) [19] (Figure 6) (DOTA = (1,4,7,10-tetraazacyclododecane-1,4,7,10-tetrayl)tetraacetic acid), having been described before to be potent agents efficiently targeting the GRPR and NPY(Y_1_)R, were synthesized in yields of 7% and 45%, respectively. These ligands, addressing only one of the target receptors types, served as monomeric reference compounds for the following in vitro evaluations.

### 2.2. ^68^Ga-Radiolabeling, log_D_ and Stability Determination of Peptide Heterodimers [^68^Ga]***22***−[^68^Ga]***26*** and Monomeric Reference Peptides [^68^Ga]***27*** and [^68^Ga]***28***

The heterobivalent ligands **22**−**26** and the reference substances **27** and **28** were in the following radiolabeled with ^68^Ga^3+^. The ^68^Ga^3+^ was obtained via elution of an itG or Eckert & Ziegler IGG100 ^68^Ge/^68^Ga generator system. After adjusting the pH of the solution to 3.5 to 4.0, the NODA-GA-comprising HBPLs **22**−**26** were incubated at 40–45 °C for 10 min. The DOTA-comprising reference compounds **27** and **28** were reacted at 99 °C under otherwise identical conditions. The ^68^Ga-labeled products [^68^Ga]**22**−[^68^Ga]**26**, [^68^Ga]**27** and [^68^Ga]**28** were obtained in radiochemical yields and purities of 95−99% (Figure S1A) as well as non-optimized molar activities of 10−15 GBq/µmol (used for in vitro assays and obtained by using an itG generator system) or 40−46 GBq/µmol (used for in vivo evaluations, obtained by using an Eckert & Ziegler IGG100 generator system), starting from 110−150 or 420–460 MBq of ^68^Ga^3+^, respectively.

Regarding a favorable in vivo biodistribution of the radioligands, the log*_D_* of the HBPLs should be in a comparable range as that of the lead peptide monomers as we and others were able to show before that a high lipophilicity negatively influences tumor uptake, organ distribution, and unspecific background accumulation, resulting in a limited usefulness of the radiopeptides for tumor visualization [23,24,25]. Consequently, we determined the log*_D_* of the developed HBPLs [^68^Ga]**22**−[^68^Ga]**26** in comparison to the monomeric reference peptides [^68^Ga]**27** and [^68^Ga]**28** via the distribution coefficient of the respective radiotracer between phosphate buffer and 1-octanol. The results of these evaluations are depicted in Figure S2. The results showed a comparatively high hydrophilicity for all of the tested substances (−1.857 ± 0.054 for [^68^Ga]**27**, −1.982 ± 0.162 for [^68^Ga]**28**, −1.569 ± 0.111 for [^68^Ga]**22**, −1.527 ± 0.109 for [^68^Ga]**23**, −1.672 ± 0.086 for [^68^Ga]**24**, −1.550 ± 0.114 for [^68^Ga]**25** and −1.518 ± 0.089 for [^68^Ga]**26**). This indicates that the in vivo pharmacokinetics of the developed HBPLs [^68^Ga]**22**−[^68^Ga]**26** should, in terms of hydrophilicity, be similar to that of the parent monomeric radiopeptides [^68^Ga]**27** and [^68^Ga]**28**.

Besides lipophilicity, the stability of peptidic radioligands is an important parameter regarding their applicability for in vivo imaging. Thus, the stability of the ^68^Ga-labeled HBPLs [^68^Ga]**22**−[^68^Ga]**26** and the reference compounds [^68^Ga]**27** and [^68^Ga]**28** was determined in human serum. Typical radio-HPLC chromatograms for each substance (obtained after 90 min incubation with human serum) are depicted in Figure S1B. All of the tested compounds were stable over the testing period of 90 min, showing only a negligible degradation after this time: 3 ± 0.2% for [^68^Ga]**27**, 4 ± 0.8% for [^68^Ga]**28**, 2 ± 1.6% for [^68^Ga]**22**, 1 ± 0.5% for [^68^Ga]**23**, no observable fragmentation for [^68^Ga]**24**, 2 ± 0.3% for [^68^Ga]**25** and 2 ± 0.5% for [^68^Ga]**26**. From the serum stability point of view—which can however only give a rough estimation of stability under in vivo conditions [26]—all of the radioligands are applicable for in vivo tumor imaging with PET/computed tomography (CT).

### 2.3. In Vitro Cell Uptake Studies: Tumor Cell Uptake of [^68^Ga]***22***−[^68^Ga]***26*** in Comparison to the Reference Peptides [^68^Ga]***27*** and [^68^Ga]***28*** in Different Human Breast Cancer Cell Lines

In the following, we intended to determine if we could observe an independent binding of both peptide parts of the HBPLs to both target receptor types, being the prerequisite for improved/more likely tumor uptake (→ Figure 1). This can be achieved by tumor cell uptake studies of the radiotracers as it was shown before for radiolabeled somatostatin analogs that the in vitro cell uptake directly correlates to in vivo tumor uptakes [27], demonstrating the relevance of such in vitro tumor cell uptake studies.

The human breast cancer cell line T-47D was described to express both the GRPR [9,28] as well as the NPY(Y_1_)R [29,30] (where β-estradiol in the medium increases NPY(Y_1_)R-expression) and thus should be the ideal cell line to determine if both parts of the developed HBPLs bind to their respective receptor and if a synergistic effect of peptide heterodimerization on tumor cell uptake can be achieved. Of course, it would also have been feasible to use different cells lines expressing either the GRPR or the NPY(Y_1_)R to demonstrate that both peptides of the HBPLs are still able to address their respective target receptor, but a cell line expressing both receptors concomitantly is far more advantageous to showcase the potential beneficial effects of heterodimerization and to determine the part each of the peptides contributes to tumor cell uptake in case of a concomitant receptor expression.

**Figure 7:**
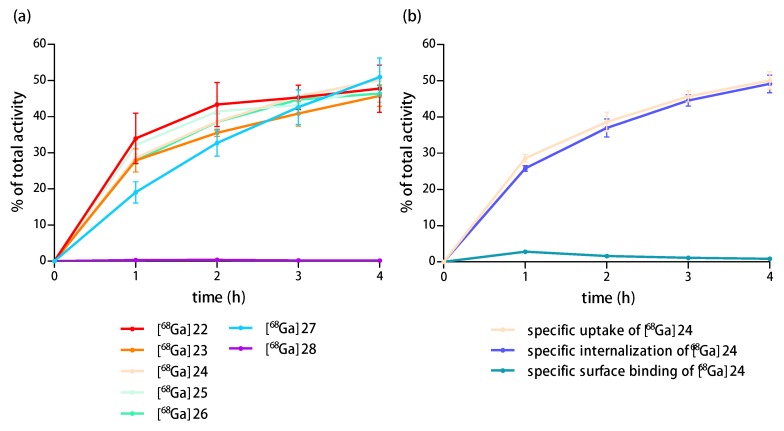
(**a**) Specific cell uptake of [^68^Ga]**22**−[^68^Ga]**26**, [^68^Ga]**27** and [^68^Ga]**28** in T-47D cells over 4 h of incubation; (**b**) Uptake of [^68^Ga]**24** in the same cells, differentiated by overall uptake, internalization, and surface binding.

Thus, we first determined the uptake of the HBPLs [^68^Ga]**22**−[^68^Ga]**26** in comparison to the peptide monomers [^68^Ga]**27** and [^68^Ga]**28** in T-47D cells. The results of the cell uptake studies of the radioligands are shown in Figure 7a (overall specific cell uptake of [^68^Ga]**22**−[^68^Ga]**26**, [^68^Ga]**27** and [^68^Ga]**28**) and Figure 7b (uptake of [^68^Ga]**24**, differentiated by overall uptake, internalization and surface binding; the results for the other tested radioligands were comparable and can be found in the Supplementary Materials in Figures S3–S7).

As expected, a high and constant specific uptake of the monovalent GRPR-specific peptide [^68^Ga]**27** of 51.0 ± 5.2% was observed into the T-47D cells after 4 h. Also, the HBPLs [^68^Ga]**22**−[^68^Ga]**26** demonstrated a comparably high specific uptake into these cells of 47.8 ± 6.5%, 45.8 ± 2.9%, 50.1 ± 2.4%, 48.2 ± 2.8% and 46.4 ± 2.4%, respectively, after the same time. Interestingly, the monovalent, NPY(Y_1_)-binding peptide [^68^Ga]**28** showed no specific uptake (0.2 ± 0.03%). This means that the uptake of the HBPLs [^68^Ga]**22**−[^68^Ga]**26** into the T-47D cells is exclusively mediated by the GRPR.

These results indicate that the heterodimerization and the resulting significant chemical modification of the peptides as well as the compounds’ complexity and size do not affect the GRPR-specific tumor cell uptake of the HBPLs compared to the monomeric reference [^68^Ga]**27**. Also, the molecular design of the heterobivalent ligands comprising linkers of different length and rigidity does not seem to have a significant effect on the GRPR-mediated cellular uptake of the respective HBPL radiotracer.

In the following, we tested the uptake of the peptide monomers [^68^Ga]**27** and [^68^Ga]**28** in three further standard cell lines of breast cancer: BT-474, MCF-7 and MDA-MB-231 cells. Of these, the BT-474 cells were also described to express both the GRPR [31] and the NPY(Y_1_)R [31], whereas MCF-7 cells were described to be NPY(Y_1_)R positive [30,32] but expressing the GRPR only to a low extent [9] and MDA-MB-231 cells were described to be GRPR positive [33] but expressing the NPY(Y_1_)R to a low extent [29,30]. The results of these experiments can be found in the Supplementary Materials (Figures S8–S10) and demonstrated contrary to the expectations only negligible uptakes of both peptide monomers in all cell lines.

As the HBPLs [^68^Ga]**22**−[^68^Ga]**26** and the respective monomer [^68^Ga]**27** were however shown to be efficiently taken up by GRPR-positive T-47D cells, these results indicate that the GRPR is present to an only low amount on BT-474, MCF-7, and MDA-MB-231 cells. Concerning the missing uptake of [^68^Ga]**28** into all tested cell lines, three explanations are possible: (i) The NPY(Y_1_)R is present on at least some of the cells but the peptide sequence [Lys^4^,Trp^5^,Nle^7^]BVD_15_, being the NPY(Y_1_)R-targeting sequence in monomer [^68^Ga]**28** as well as the HBPLs [^68^Ga]**22**−[^68^Ga]**26**, is not able to efficiently address the NPY(Y_1_)R; (ii) The NPY(Y_1_)R is expressed in such low amounts that the tested radioligands cannot be efficiently taken up by the cells by this receptor; or (iii) The molar activities of the tested radioligands were too low and a significant self-blocking of the NPY(Y_1_)R-mediated uptake took place, preventing the cell uptake.

In order to determine if the NPY(Y_1_)R is actually present on T-47D, MCF-7 and BT-474 cells and if the molar activity of monomer [^68^Ga]**28** and the HBPLs [^68^Ga]**22**−[^68^Ga]**26** prevented their cellular uptake via the NPY(Y_1_)R, the cell uptake studies were repeated using commercially available ^125^I-PYY, binding the NPY(Y_1_)R [29] and exhibiting a high molar activity of 81.4 GBq/µmol. In these experiments, an about 100-fold lower amount of substance was used for the cell uptake studies compared to the ^68^Ga-labeled ligands ([^68^Ga]**22**−[^68^Ga]**26**, [^68^Ga]**27** and [^68^Ga]**28**: 0.37−0.40 pmol per 1.5 × 10^6^ cells; ^125^I-PYY: 0.003−0.005 pmol per 1.5 × 10^6^ cells) in order to exclude the eventuality of self-blocking. In all of the three tested cell lines, the specific uptake of ^125^I-PYY showed to be negligibly low (between 0.2 and 2.5% of the total activity applied), confirming that the target NPY(Y_1_) receptor might be present on the cells but if so, then only to a very low amount, being too low to give useful results in the cell uptake studies.

Furthermore, this low receptor density prevents a successful determination of the receptor affinity of the developed radioligands. This is in contrast to other studies, using identically treated MCF-7 cells to determine NPY(Y_1_)R affinities of newly developed NPY(Y_1_)R-affine radioligands [9,19]. We could however not reproduce these results using MCF-7 cells of different suppliers due to the observed low expression of NPY(Y_1_)R on the cells.

As it was described that MCF-7 cells can express the target NPY(Y_1_)R to a much higher extent in vivo than under in vitro conditions [34], this might also be the case for the T-47D cell line, expressing besides the NPY(Y_1_)R also the for our scientific question important GRPR. Thus, we in the following performed initial in vivo evaluations of HBPL [^68^Ga]**24**, showing highly promising results in the preceding evaluations. Of the developed HBPLs, [^68^Ga]**24** showed the highest stability and hydrophilicity as well as a slightly higher tumor cell uptake than the other analogs in vitro and thus represents a potent representative of the developed HBPLs for a following proof-of-concept in vivo evaluation.

### 2.4. Proof-of-Concept: Evaluation of HBPL [^68^Ga]***24*** and Its Scrambled Analogs [^68^Ga]***24a*** and [^68^Ga]***24b*** via In Vivo PET/CT Imaging in T-47D-Bearing Nude Mice and Ex Vivo Biodistribution

To investigate the in vivo pharmacokinetic properties of the most potent HBPL [^68^Ga]**24**, we performed small animal PET/CT imaging studies in T-47D tumor-bearing immunodeficient mice. Likewise, also the scrambled variants of [^68^Ga]**24**, [^68^Ga]**24a** (PESIN_scrambled_ combined with [Lys^4^(aminooxy),Trp^5^,Nle^7^]BVD_15_) and [^68^Ga]**24b** (PESIN combined with [Lys^4^(aminooxy),Trp^5^,Nle^7^]BVD_15,scrambled_), were evaluated under the same conditions for comparison.

By evaluating these radioligands under the same conditions, the proportion of both peptide binders on the uptake of [^68^Ga]**24** and also the receptor-specificity of the uptake of the radioligand via both receptors should be determined. The evaluation of the partly scrambled monovalent analogs instead of the monomeric peptides, which would also have been possible, exhibits the advantage that all evaluated radioligands show a similar pharmacokinetic distribution and also a similar possible degradation pattern. Using the monovalent peptides bombesin or NPY/BVD for receptor blocking (to show the receptor specificity of both peptide parts of [^68^Ga]**24** and to determine the contribution of both peptide parts of the HBPL to overall tumor uptake) might have resulted in an incomplete blocking of the respective receptor as the GRPR-affine bombesin as well as the NPY(Y_1_)R-affine peptides NPY and BVD are known for their limited in vivo stability.

**Figure 8:**
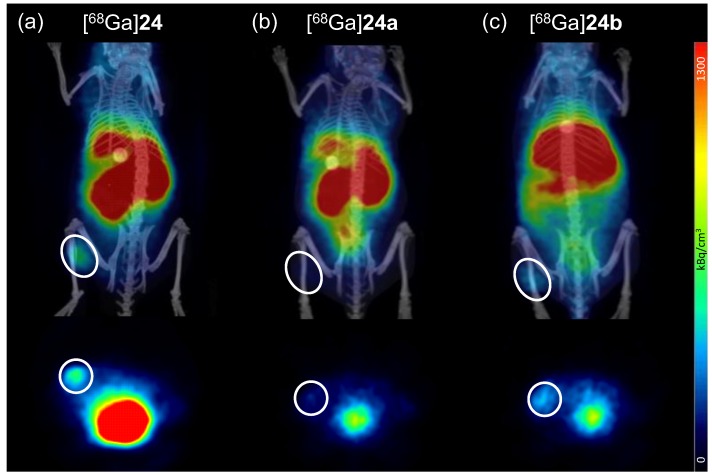
Representative small animal positron emission tomography/computed tomography (PET/CT) imaging results for [^68^Ga]**24** (**a**); [^68^Ga]**24a** (**b**) and [^68^Ga]**24b** (**c**). The images show coronal (**upper row**) and transaxial (**lower row**) slices for all tracers at 37.5 min p.i. The same scaling was used for all images and 4 animals were examined per radioligand. The tumor is encircled in each image.

For the PET/CT imaging studies, 5.5–8.0 MBq of the respective ^68^Ga-radioligand were administered via the lateral tail vein under isoflurane anesthesia to the tumor-bearing animals. Directly after completion of the diagnostic scans, the animals were sacrificed, the organs were collected and measured in a gamma-counter. The results of these in vivo PET/CT imaging studies and the ex vivo biodistribution data are given in Figure 8 and Table S1.

As can easily be seen from the in vivo PET/CT imaging as well as the ex vivo biodistribution data, all developed ligands showed a rather high kidney and liver uptake. Thus, further improvements in ligand design must be carried out to result in clinically relevant imaging agents for improved breast cancer visualization with PET/CT.

Compared to the bispecific ligand [^68^Ga]**24** with which the tumor can easily be delineated via PET/CT, the scrambled analogs [^68^Ga]**24a** and [^68^Ga]**24b** showed a considerably less efficient tumor visualization ability (Figure 8).

**Figure 9:**
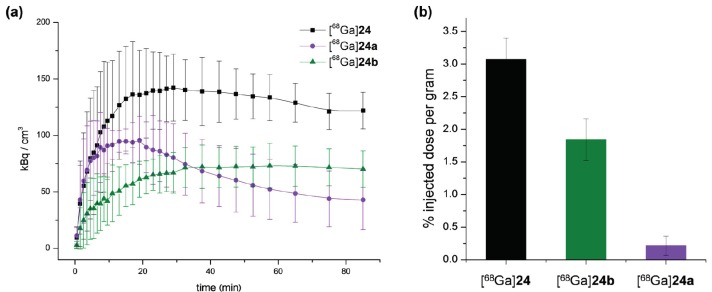
Absolute tumor uptakes as determined by in vivo PET imaging as well as ex vivo biodistribution experiments (*n* = 4). (**a**) Time-activity-curves are depicted, showing tumor uptakes over the course of PET imaging for [^68^Ga]**24** (black), [^68^Ga]**24a** (purple) and [^68^Ga]**24b** (green); (**b**) Tumor uptakes (% injected dose per gram) are shown for [^68^Ga]**24** (black), [^68^Ga]**24a** (purple) and [^68^Ga]**24b** (green) as determined by ex vivo biodistribution directly after the completed diagnostic scans.

This results from a lower absolute tumor uptake for [^68^Ga]**24a** and [^68^Ga]**24b** compared to [^68^Ga]**24** over the course of PET imaging (Figure 9a, SUVs_(85min)_ of 122.03 ± 16.27 kBq/cm^3^ for [^68^Ga]**24**, 42.95 ± 26.38 kBq/cm^3^ for [^68^Ga]**24a** and 70.29 ± 16.09 kBq/cm^3^ for [^68^Ga]**24b** were observed), which was also confirmed in the ex vivo biodistribution experiments (Figure 9b).

This indicates that both parts of HBPL [^68^Ga]**24** contributed to in vivo tumor uptake and that its uptake into the tumor was GRPR- and also NPY(Y_1_)R-specific.

As can be observed from the time-activity-curves obtained by PET imaging (Figure 9a), those ligands comprising an intact variant of the GRPR-targeting ligand BBN_7–14_ showed a stable plateau phase in tumor accumulation ([^68^Ga]**24b**) or only a slight decrease ([^68^Ga]**24**) whereas [^68^Ga]**24a**, comprising a scrambled variant of BBN_7–14_, shows a surge in tumor uptake followed by a decline of activity in the tumor. This might be attributable to the limited stability of BVD analogs under in vivo conditions, which has been described before [35] and might result in radioligand metabolization during imaging. If then the GRPR-binding peptide is still able to bind to its target receptor, the tumor uptake nevertheless remains largely stable due to a GRPR-mediated uptake. However, if only a scrambled variant of BBN_7–14_ is present, no receptor-affine peptide binder remains for continuous radiotracer uptake into the tumor, resulting in an overall decrease in tumor accumulation.

Further, high and considerably improved tumor-to-background ratios could be achieved for [^68^Ga]**24** (tumor-to-muscle: 11.81 ± 1.83, tumor-to-blood: 2.72 ± 0.43) compared to [^68^Ga]**24a** (tumor-to-muscle: 1.07 ± 1.14, tumor-to-blood: 0.50 ± 0.47) and [^68^Ga]**24b** (tumor-to-muscle: 3.83 ± 0.80, tumor-to-blood: 0.91 ± 0.24) as determined by ex vivo biodistribution at 130 min p.i. This indicates that both peptide parts of the HBPL contributed to tumor uptake and thus tumor visualization.

In summary, we were able to show here for the first time that the general concept to assemble a GRPR- and a NPY(Y_1_)R-affine peptide to one combined radioligand is in general feasible regarding a contribution of both peptides of the HBPL to in vivo tumor uptake and thus is beneficial with regard to overall tumor uptake and tumor visualization probability compared to the monospecific agents.

## 3. Materials and Methods

**General.** All commercially available chemicals were of analytical grade and were used without further purification. Resins for solid phase-based syntheses, PyBOP (Benzotriazole-1-yl-oxy-*tris*-pyrrolidino-phosphonium hexafluorophosphate), Fmoc-protected standard amino acids as well as *bis*-Boc-aminooxy acetic acid were purchased from NovaBiochem (Darmstadt, Germany). SFB (Succinimidyl-*p*-formyl-benzoate) was synthesized according to a published procedure [36]. Fmoc-ACMP-OH (Fmoc-4-amino-1-carboxymethyl-piperidine) was obtained from Iris Biotech (Marktredwitz, Germany), respectively. Fmoc-l-Lys(Boc_2_-Aoa)-OH, mono-Fmoc ethylene diamine hydrochloride, HBTU (*O*-(Benzotriazol-1-yl)-*N*,*N*,*N*′,*N*′-tetramethyluronium hexafluorophosphate), Tracepur water and *N*,*N*-bis(*N*′-Fmoc-3-aminopropyl)-glycine potassium hemisulfate ((Fmoc-NH-Propyl)_2_-Gly-OH) were purchased from Iris Biotech, SigmaAldrich (Schnelldorf, Germany), Carl Roth (Karlsruhe, Germany), VWR (Bruchsal, Germany) and PolyPeptide (Strasbourg, France), respectively. NODA-GA-(*t*Bu)_3_ and DOTA-(*t*Bu)_3_ were obtained from CheMatech (Dijon, France).

*Bis*-amines **7**–**11** and *bis*-aldehydes **12**–**16** were synthesized according to published procedures [22] with minor modifications of the synthesis protocols. Details of these syntheses can be found in the supplementary information.

Unless otherwise stated, the coupling reactions during solid phase-based syntheses were usually carried out in DMF for 30 min using 4 eq. of acid, 3.9 eq. of HBTU as coupling reagent and 4 eq. of DIPEA (*N*,*N*-Diisopropylethylamine) as base. Fmoc protecting groups were removed using 50% (*v*/*v*) piperidine in DMF.

For analytical and semipreparative HPLC chromatography, Dionex UltiMate 3000 systems equipped with a Chromolith Performance (RP-18e, 100-4.6 mm, Merck, Darmstadt, Germany) and a Chromolith SemiPrep (RP-18e, 100-10 mm, Merck) column were used, operated with a flow rate of 4 mL/min and H_2_O + 0.1% TFA and MeCN + 0.1% TFA as eluents. For radio-analytical HPLC chromatography, a Dionex UltiMate 3000 system equipped with a Chromolith Performance (RP-18e, 100-4.6 mm, Merck) column and a GabiStar radioactivity detector (Raytest, Straubenhardt, Germany) or an Agilent 1200 system equipped with a Chromolith Performance (RP-18e, 100-4.6 mm, Merck) column and a GabiStar radioactivity detector (Raytest) were used and operated with a flow rate of 4 mL/min and H_2_O + 0.1% TFA and MeCN + 0.1% TFA as eluents. MALDI (Matrix-Assisted Laser Desorption/Ionization) spectra were obtained with a Bruker Daltonics Microflex spectrometer (Bremen, Germany).

The human breast cancer cell lines T-47D, MDA-MB-231 and MCF-7 were purchased from SigmaAldrich (Schnelldorf, Germany), whereas the cell line BT-474 was obtained from the Leibniz-Institute DSMZ (Braunschweig, Germany). Dulbecco’s Modified Eagle Medium (DMEM), RPMI-1640 medium, 200 mM L-glutamine, 0.05% trypsin/EDTA and 0.25% trypsin/EDTA were purchased from Life Technologies. Fetal calf serum (FCS) was obtained from GE Healthcare Life Sciences and phosphate buffered saline (PBS) as well as β-estradiol were purchased from Sigma Aldrich. Bovine serum albumin (BSA) was purchased from CarlRoth (Karlsruhe, Germany).

The human ^125^I-labeled NPY(Y_1_)-binding peptide [^125^I]-Peptide-YY was obtained from PerkinElmer in a molar activity of 81.4 GBq/µmol. The γ-counter used was a 2480 WIZARD^2^ system (PerkinElmer, Rodgau, Germany).

For the in vivo evaluations, five week old female Fox Chase SCID mice were obtained from Janvier and implanted with estradiol pellets (0.36 mg/60 days; obtained from Innovative Research of America) one week prior to tumor cell inoculation. Tumor cells were inoculated using Matrigel basal membrane matrix with reduced growth factor (obtained from VWR). For PET/CT measurements, a small animal Albira II PET/SPECT/CT system (Bruker, Eggenstein-Leopoldshafen, Germany) was used.

**Synthesis of aminooxy-PESIN (1) and aminooxy-PESIN_scrambled_ (5).** The peptides were synthesized on solid support by standard Fmoc solid-phase peptide synthesis using a commercially available standard Rink amide MBHA resin, HBTU as coupling reagent, standard *N*_α_-Fmoc-amino acids, *N*_ω_-Fmoc-PEG_4_-OH and *bis*-Boc-aminooxy acetic acid. All amino acids (apart from *bis*-Boc-aminooxy acetic acid which was reacted for 60 min) were coupled within 30 min. The crude aminooxy-modified peptides were cleaved from the solid support using a mixture of TFA:TIS:H_2_O of 95:2.5:2.5 (*v*/*v*) for 60 min, suspended in diethyl ether and purified by semipreparative HPLC.

**1** was purified using a gradient of 15–30% MeCN + 0.1% TFA in 8 min (R_t_ = 6.35 min) and isolated as white solid after lyophilization in yields of 27% (85.0 mg; 67.4 µmol). MALDI-MS (*m*/*z*) using 2,5-dihydroxybenzoic acid as matrix substance for [M + H^+^]^+^ (calculated): 1260.23 (1260.46); [M + Na^+^]^+^ (calculated): 1282.21 (1282.62); [M + K^+^]^+^ (calculated): 1298.16 (1298.60). MALDI-MS (*m*/*z*) using α-cyano-4-hydroxycinnamic acid as matrix substance for [M + H^+^]^+^ (calculated): 1260.63 (1260.46); [M + Na^+^]^+^ (calculated): 1282.56 (1282.62); [M + K^+^]^+^ (calculated): 1298.54 (1298.60).

**5** was purified using a gradient of 10–60% MeCN + 0.1% TFA in 8 min (R_t_ = 5.42 min) and isolated as white solid after lyophilization in yields of 22% (28.1 mg, 22.2 µmol). MALDI-MS (*m*/*z*) using α-cyano-4-hydroxycinnamic acid as matrix substance for [M + H^+^]^+^ (calculated): 1260.81 (1260.46); [M + Na^+^]^+^ (calculated): 1282.84 (1282.62); [M + K^+^]^+^ (calculated): 1298.78 (1298.60).

**Synthesis of [Lys^4^(aminooxy),Trp^5^,Nle^7^]BVD15 (2) and [Lys^4^(aminooxy),Trp^5^,Nle^7^]BVD15_scrambled_ (6).** The peptides were synthesized on solid support by standard Fmoc solid-phase peptide synthesis using a commercially available Rink amide MBHA resin, HBTU as coupling reagent, standard *N*_α_-Fmoc-amino acids and *N*_α_-Fmoc-l-Lys(Boc_2_-Aoa)-OH. The crude aminooxy-modified peptides were cleaved from the solid support using a mixture of TFA:TIS:H_2_O of 95:2.5:2.5 (*v*/*v*) for 90 min, suspended in diethyl ether and purified by semipreparative HPLC.

**2** was purified using a gradient of 10–60% MeCN + 0.1% TFA in 8 min (R_t_ = 3.71 min) and isolated as white solid after lyophilization in yields of 35% (114.2 mg; 86.7 µmol). MALDI-MS (*m*/*z*) using 2,5-dihydroxybenzoic acid as matrix substance for [M + H^+^]^+^ (calculated): 1317.02 (1317.54); [M + Na^+^]^+^ (calculated): 1339.13 (1339.74); [M + K^+^]^+^ (calculated): 1355.03 (1355.71). MALDI-MS (*m*/*z*) using α-cyano-4-hydroxycinnamic acid as matrix substance for [M + H^+^]^+^ (calculated): 1317.89 (1317.54); [M + Na^+^]^+^ (calculated): 1339.89 (1339.74); [M + K^+^]^+^ (calculated): 1355.86 (1355.71).

**6** was purified using a gradient of 10–50% MeCN + 0.1% TFA in 8 min (R_t_ = 4.31 min) and isolated as white solid after lyophilization in yields of 20% (26.0 mg; 19.7 µmol). MALDI-MS (*m*/*z*) using α-cyano-4-hydroxycinnamic acid as matrix substance for [M + H^+^]^+^ (calculated): 1317.37 (1317.54); [M + Na^+^]^+^ (calculated): 1339.38 (1339.74).

**Synthesis of bombesin (3).** The peptide was synthesized on solid support by standard Fmoc solid-phase peptide synthesis using a commercially available standard Rink amide resin, HBTU as coupling reagent and standard *N*_α_-Fmoc-amino acids. All amino acids were coupled within 35 min. The crude peptide was cleaved from the solid support using a mixture of TFA:TIS:H_2_O of 95:2.5:2.5 (*v*/*v*) for 90 min, suspended in diethyl ether and purified by semipreparative HPLC using a gradient of 15–60% MeCN + 0.1% TFA in 5.5 min (R_t_ = 3.40 min) and isolated as white solid after lyophilization in yields of 22% (35.2 mg, 21.6 µmol). MALDI-MS (*m*/*z*) using α-cyano-4-hydroxycinnamic acid as matrix substance for [M + H^+^]^+^ (calculated): 1618.56 (1618.82); [M + Na^+^]^+^ (calculated): 1640.55 (1640.81); [M + K^+^]^+^ (calculated): 1656.57 (1656.78).

**Synthesis of [Lys^4^,Trp^5^,Nle^7^]BVD15 (4).** The peptide was synthesized on solid support by standard Fmoc solid-phase peptide synthesis using a commercially available standard Rink amide resin, HBTU as coupling reagent and standard *N*_α_-Fmoc-amino acids. All amino acids were coupled within 30 min. The crude peptide was cleaved from the solid support using a mixture of TFA:TIS:H_2_O of 95:2.5:2.5 (*v*/*v*) for 120 min, suspended in diethyl ether and purified by semipreparative HPLC using a gradient of 10–60% MeCN + 0.1% TFA in 5.5 min (R_t_ = 3.05 min) and isolated as white solid after lyophilization in yields of 40% (49.2 mg; 39.5 µmol). MALDI-MS (*m*/*z*) using α-cyano-4-hydroxycinnamic acid as matrix substance for [M + H^+^]^+^ (calculated): 1244.63 (1244.73); [M + Na^+^]^+^ (calculated): 1266.65 (1266.72); [M + K^+^]^+^ (calculated): 1282.60 (1282.69).

**General synthesis of NODA-GA-PESIN-aldehydes (17−21) and NODA-GA-PESIN_scrambled_-aldehyde (19a).** To a solution of the respective branched NODA-GA-*bis*-aldehyde (**12**−**16**) in H_2_O + 0.1% TFA (250−500 µL) was added a solution of aminooxy-PESIN (**1**) or aminooxy-PESIN_scrambled_ (**5**) (0.7 eq.) in H_2_O + 0.1% TFA (250−500 µL). The pH of the solutions was adjusted to 4.0–4.6 by addition of phosphate buffer (0.1 M, pH 7.2, ~150 µL) and the reaction progress was monitored by analytical HPLC. The reactions were found to be finished within 5 min and the products were purified by semipreparative HPLC. The products were isolated as white solids after lyophilization. Gradients used for HPLC purification and synthesis yields for each compound are given below.

**17**: gradient: 20–45% MeCN + 0.1% TFA in 5 min (R_t_ = 4.45 min), yield: 47%. MALDI-MS (*m*/*z*) using α-cyano-4-hydroxycinnamic acid as matrix substance for [M + H^+^]^+^ (calculated): 2427.33 (2427.22); [M + Na^+^]^+^ (calculated): 2449.30 (2449.21); [M + K^+^]^+^ (calculated): 2465.49 (2465.18). MALDI-MS (*m*/*z*) using 2,5-dihydroxybenzoic acid as matrix substance for [M + H^+^]^+^ (calculated): 2426.69 (2427.22); [M + Na^+^]^+^ (calculated): 2448.99 (2449.21); [M + K^+^]^+^ (calculated): 2464.93 (2465.18). MALDI-MS (*m*/*z*) using sinapic acid as matrix substance for [M + H^+^]^+^ (calculated): 2427.38 (2427.22); [M + Na^+^]^+^ (calculated): 2449.25 (2449.21).

**18**: gradient: 25–45% MeCN + 0.1% TFA in 5 min (R_t_ = 3.76 min), yield: 51%. MALDI-MS (*m*/*z*) using α-cyano-4-hydroxycinnamic acid as matrix substance for [M + H^+^]^+^ (calculated): 2717.48 (2717.37); [M + Na^+^]^+^ (calculated): 2739.21 (2739.36); [M + K^+^]^+^ (calculated): 2755.49 (2755.33). MALDI-MS (*m*/*z*) using 2,5-dihydroxybenzoic acid as matrix substance for [M + H^+^]^+^ (calculated): 2717.18 (2717.37); [M + Na^+^]^+^ (calculated): 2739.60 (2739.36); [M + K^+^]^+^ (calculated): 2755.13 (2755.33).

**19**: gradient: 25–45% MeCN + 0.1% TFA in 5 min (R_t_ = 4.26 min), yield: 43%. MALDI-MS (*m*/*z*) using α-cyano-4-hydroxycinnamic acid as matrix substance for [M + H^+^]^+^ (calculated): 2921.63 (2921.50); [M + Na^+^]^+^ (calculated): 2943.64 (2943.49); [M + K^+^]^+^ (calculated): 2959.46 (2959.47). MALDI-MS (*m*/*z*) using 2,5-dihydroxybenzoic acid as matrix substance for [M + H^+^]^+^ (calculated): 2921.63 (2921.50); [M + Na^+^]^+^ (calculated): 2943.34 (2943.49); [M + K^+^]^+^ (calculated): 2959.57 (2959.47).

**19a** (PESIN scrambled): gradient: 25–45% MeCN + 0.1% TFA in 5.5 min (R_t_ = 4.18 min), yield: 44%. MALDI-MS (*m*/*z*) using α-cyano-4-hydroxycinnamic acid as matrix substance for [M + H^+^]^+^ (calculated): 2921.59 (2921.50); [M + Na^+^]^+^ (calculated): 2943.56 (2943.49); [M + K^+^]^+^ (calculated): 2959.52 (2959.47).

**20**: gradient: 20–45% MeCN + 0.1% TFA in 5 min (R_t_ = 3.93 min), yield: 58%. MALDI-MS (*m*/*z*) using α-cyano-4-hydroxycinnamic acid as matrix substance for [M + H^+^]^+^ (calculated): 2707.24 (2707.41); [M + Na^+^]^+^ (calculated): 2729.30 (2729.40); [M + K^+^]^+^ (calculated): 2745.58 (2745.37). MALDI-MS (*m*/*z*) using 2,5-dihydroxybenzoic acid as matrix substance for [M + H^+^]^+^ (calculated): 2706.98 (2707.41); [M + Na^+^]^+^ (calculated): 2728.88 (2729.40); [M + K^+^]^+^ (calculated): 2744.78 (2745.37).

**21**: gradient: 25–45% MeCN + 0.1% TFA in 5 min (R_t_ = 2.85 min), yield: 55%. MALDI-MS (*m*/*z*) using α-cyano-4-hydroxycinnamic acid as matrix substance for [M + H^+^]^+^ (calculated): 2987.39 (2987.60); [M + Na^+^]^+^ (calculated): 3009.40 (3009.59); [M + K^+^]^+^ (calculated): 3025.80 (3025.56). MALDI-MS (*m*/*z*) using 2,5-dihydroxybenzoic acid as matrix substance for [M + H^+^]^+^ (calculated): 2987.03 (2987.60). MALDI-MS (*m*/*z*) using sinapic acid as matrix substance for [M + H^+^]^+^ (calculated): 2987.81 (2987.60).

**General synthesis of heterobivalent ligands 22−26 and scrambled analogs 24a–c.** To a solution of the respective NODA-GA-PESIN-aldehyde (**17**−**21** or **19a**) in H_2_O + 0.1% TFA (250−500 µL) was added a solution of **2** or **6** (3 eq.) in H_2_O + 0.1% TFA (250−500 µL). The pH of the solutions was adjusted to 4.0–4.6 by addition of phosphate buffer (0.1 M, pH 7.2, ~150 µL) and the reaction progress was monitored by analytical HPLC. The reactions were found to be finished within 5 min and the products were purified by semipreparative HPLC. The products were isolated as white solids after lyophilization. Gradients used for HPLC purification and synthesis yields for each compound are given below.

**22**: gradient: 25–50% MeCN + 0.1% TFA in 5 min (R_t_ = 3.19 min), yield: 82%. MALDI-MS (*m*/*z*) using α-cyano-4-hydroxycinnamic acid as matrix substance for [M + H^+^]^+^ (calculated): 3728.21 (3728.29); [M + Na^+^]^+^ (calculated): 3750.49 (3750.28). MALDI-MS (*m*/*z*) using 2,5-dihydroxybenzoic acid as matrix substance for [M + H^+^]^+^ (calculated): 3728.71 (3728.29); [M + Na^+^]^+^ (calculated): 3750.76 (3750.28).

**23**: gradient: 25–45% MeCN + 0.1% TFA in 5 min (R_t_ = 3.65 min), yield: 79%. MALDI-MS (*m*/*z*) using α-cyano-4-hydroxycinnamic acid as matrix substance for [M + H^+^]^+^ (calculated): 4018.99 (4018.61); [M + Na^+^]^+^ (calculated): 4040.69 (4040.60). MALDI-MS (*m*/*z*) using 2,5-dihydroxybenzoic acid as matrix substance for [M + H^+^]^+^ (calculated): 4018.95 (4018.61); [M + Na^+^]^+^ (calculated): 4040.02 (4040.60).

**24**: gradient: 25–45% MeCN + 0.1% TFA in 6 min (R_t_ = 3.86 min), yield: 66%. MALDI-MS (*m*/*z*) using α-cyano-4-hydroxycinnamic acid as matrix substance for [M + H^+^]^+^ (calculated): 4222.98 (4222.87). MALDI-MS (*m*/*z*) using 2,5-dihydroxybenzoic acid as matrix substance for [M + H^+^]^+^ (calculated): 4222.48 (4222.87); [M + Na^+^]^+^ (calculated): 4244.96 (4244.86); [M + K^+^]^+^ (calculated): 4260.51 (4260.97). MALDI-MS (*m*/*z*) using sinapic acid as matrix substance for [M + H^+^]^+^ (calculated): 4222.87 (4222.87).

**24a** (PESIN scrambled): gradient: 25–45% MeCN + 0.1% TFA in 6 min (R_t_ = 3.55 min), yield: 49%. MALDI-MS (*m*/*z*) using α-cyano-4-hydroxycinnamic acid as matrix substance for [M + H^+^]^+^ (calculated): 4222.54 (4222.87). MALDI-MS (*m*/*z*) using 2,5-dihydroxybenzoic acid as matrix substance for [M + H^+^]^+^ (calculated): 4222.17 (4222.87); [M + Na^+^]^+^ (calculated): 4244.94 (4244.86).

**24b** ([Lys^4^,Trp^5^,Nle^7^]BVD_15_ scrambled): gradient: 25–40% MeCN + 0.1% TFA in 5.5 min (R_t_ = 4.58 min), yield: 63%. MALDI-MS (*m*/*z*) using α-cyano-4-hydroxycinnamic acid as matrix substance for [M + H^+^]^+^ (calculated): 4222.35 (4222.87). MALDI-MS (*m*/*z*) using 2,5-dihydroxybenzoic acid as matrix substance for [M + H^+^]^+^ (calculated): 4222.29 (4222.87); [M + Na^+^]^+^ (calculated): 4244.99 (4244.86); [M + K^+^]^+^ (calculated): 4260.49 (4260.97).

**24c** (PESIN and [Lys^4^,Trp^5^,Nle^7^]BVD_15_ scrambled): gradient: 25–45% MeCN + 0.1% TFA in 5.5 min (R_t_ = 4.36 min), yield: 61%. MALDI-MS (*m*/*z*) using α-cyano-4-hydroxycinnamic acid as matrix substance for [M + H^+^]^+^ (calculated): 4222.67 (4222.87); [M + K^+^]^+^ (calculated): 4260.98 (4260.97). MALDI-MS (*m*/*z*) using 2,5-dihydroxybenzoic acid as matrix substance for [M + H^+^]^+^ (calculated): 4222.48 (4222.87); [M + Na^+^]^+^ (calculated): 4244.90 (4244.86).

**25**: gradient: 25–50% MeCN + 0.1% TFA in 6 min (R_t_ = 2.76 min), yield: 75%. MALDI-MS (*m*/*z*) using α-cyano-4-hydroxycinnamic acid as matrix substance for [M + H^+^]^+^ (calculated): 4008.53 (4008.66); [M + Na^+^]^+^ (calculated): 4030.76 (4030.65); [M + K^+^]^+^ (calculated): 4046.33 (4046.76). MALDI-MS (*m*/*z*) using 2,5-dihydroxybenzoic acid as matrix substance for [M + H^+^]^+^ (calculated): 4009.12 (4008.66).

**26**: gradient: 25–45% MeCN + 0.1% TFA in 6 min (R_t_ = 2.83 min), yield: 73%. MALDI-MS (*m*/*z*) using 2,5-dihydroxybenzoic acid as matrix substance for [M + H^+^]^+^ (calculated): 4288.72 (4289.03). MALDI-MS (*m*/*z*) using sinapic acid as matrix substance for [M + H^+^]^+^ (calculated): 4289.59 (4289.03).

**Synthesis of DOTA-PESIN (27).** The peptide was synthesized on solid support by standard Fmoc solid-phase peptide synthesis using a commercially available standard Rink amide MBHA resin, HBTU as coupling reagent, standard *N*_α_-Fmoc-amino acids, and *N*_ω_-Fmoc-PEG_4_-OH. After the conjugation of the PEG_4_ linker to the peptide sequence, DOTA-(tBu)_3_ was coupled within 120 min using an excess of the synthon of 2.7 eq. together with 2.6 eq. HBTU and 4 eq. DIPEA. The crude product was cleaved from the solid support using a mixture of TFA:TIS:H_2_O of 95:2.5:2.5 (*v*/*v*) for 3 h, suspended in diethyl ether and purified by semipreparative HPLC using a gradient of 20–35% MeCN + 0.1% TFA in 8 min (R_t_ = 4.34 min) and isolated as white solid after lyophilization in yields of 7% (10.7 mg; 6.8 µmol). MALDI-MS (*m*/*z*) using 2,5-dihydroxybenzoic acid as matrix substance for [M + H^+^]^+^ (calculated): 1573.02 (1573.80); [M + Na^+^]^+^ (calculated): 1595.06 (1595.79); [M + K^+^]^+^ (calculated): 1610.94 (1611.76). MALDI-MS (*m*/*z*) using α-cyano-4-hydroxycinnamic acid as matrix substance for [M + H^+^]^+^ (calculated): 1573.75 (1573.80); [M + Na^+^]^+^ (calculated): 1595.85 (1595.79).

**Synthesis of [Lys^4^(DOTA),Trp^5^,Nle^7^]BVD_15_ (28).** The peptide was synthesized on solid support by standard Fmoc solid-phase peptide synthesis using a commercially available standard Rink amide MBHA resin, HBTU as coupling reagent, standard *N*_α_-Fmoc-amino acids and Fmoc-Lys(Mtt)-OH. After conjugation of the last amino acid, the lysine side chain Mtt-protecting group was removed with diluted TFA (TFA:DCM 1:99 (*v*/*v*)) within 2 h and DOTA-(tBu)_3_ was coupled in this position within 120 min using an excess of the synthon of 2.7 eq. together with 2.6 eq. HBTU and 4 eq. DIPEA. The crude DOTA-modified peptide was cleaved from the solid support using a mixture of TFA:TIS:H_2_O of 95:2.5:2.5 (*v*/*v*) for 3 h, suspended in diethyl ether and purified by semipreparative HPLC using a gradient of 20–25% MeCN + 0.1% TFA in 8 min (R_t_ = 2.58 min) and isolated as white solid after lyophilization in yields of 45% (73.4 mg; 45.0 µmol). MALDI-MS (*m*/*z*) using 2,5-dihydroxybenzoic acid as matrix substance for [M + H^+^]^+^ (calculated): 1630.38 (1630.91); [M + Na^+^]^+^ (calculated): 1652.59 (1652.90); [M + K^+^]^+^ (calculated): 1668.39 (1668.87). MALDI-MS (*m*/*z*) using α-cyano-4-hydroxycinnamic acid as matrix substance for [M + H^+^]^+^ (calculated): 1630.54 (1630.91). MALDI-MS (*m*/*z*) using sinapic acid as matrix substance for [M + H^+^]^+^ (calculated): 1630.53 (1630.91).

**^68^Ga-radiolabeling of NODAGA-modified peptide heterodimers (22−26) and peptide monomers 27 and 28 for in vitro evaluations**. The respective labeling precursor (10 nmol, dissolved in 10 µL of Tracepur water) was reacted with 110–150 MBq of ^68^Ga^3+^ obtained by an itG ^68^Ge/^68^Ga generator system (Garching, Germany). The generator was eluted with HCl (0.05 M, 3 mL) and the eluate was trapped on a cation exchange cartridge (Macherey-Nagel, Chromafix PS-H^+^). The ^68^Ga^3+^ was eluted from the cartridge using a NaCl solution (5 M, 1.5 mL) and the pH was adjusted to 3.5–4.0 by addition of sodium acetate solution (1.25 M, ~50 µL). After reaction for 10 min at 45 °C (**22**−**26**) or 99 °C (**27** and **28**), the reaction mixtures were analyzed by analytical radio-HPLC. The radiolabeled products were found to be 95–99% pure and obtained in molar activities of 10–15 GBq/µmol (non-optimized).

**^68^Ga-radiolabeling of NODAGA-modified peptide heterodimers (24 and 24a–c) for in vivo evaluations**. The respective labeling precursor (10 nmol, dissolved in 10 µL of Tracepur water) was reacted with 420–460 MBq of ^68^Ga^3+^ obtained by fractioned elution of an Eckert & Ziegler ^68^Ge/^68^Ga generator system (IGG100, Eckert & Ziegler, Berlin, Germany). The generator was eluted with HCl (0.1 M, 1.4 mL) and the pH was adjusted to 3.5–4.0 by addition of sodium acetate solution (1.25 M, 90–95 µL). After reaction for 10 min at 45 °C, the reaction mixtures were analyzed by analytical radio-HPLC. The radiolabeled products were found to be 95–99% pure and obtained in molar activities of 40–46 GBq/µmol (non-optimized). The pH of the radiotracer solution was adjusted to 6.0–7.0 using HEPES buffer (2.0 M, pH 8.0, 200 µL) and used for the in vivo studies.

**Determination of radiotracer lipophilicity.** The heterobivalent ligands (**22**–**26**) as well as the monomeric reference compounds (**27** and **28**) were radiolabeled with ^68^Ga as described before and 2 µL of the product solution (~65 pmol of the respective radioligand) were added to a mixture of phosphate buffer (0.05 M, pH 7.4, 800 µL) and 1-octanol (800 µL) and incubated for 5 min at ambient temperature under vigorous shaking. Both phases were separated by centrifugation and 100 µL of each phase were measured for radioactivity in a gamma-counter. From these data, the distribution coefficient log*_D_* was calculated from the following equation: log*D_o/w_* = log(cpm_o_/cpm_w_), where: cpm_o_ = activity in the 1-octanol phase [cpm] (cpm = counts per minute), cpm_w_ = activity in the aqueous phase [cpm]. These experiments were performed six times independently.

**Determination of the stability of the ligands in human serum.** The heterobivalent ligands (**22**–**26**) as well as the monomeric reference compounds (**27** and **28**) were radiolabeled with ^68^Ga as described before and 125 µL of the product solution were added to 500 µL of human serum and incubated at 37 °C. At defined time-points of 5, 15, 30, 60 and 90 min, aliquots of 75 µL of the mixture were added to 75 µL of ethanol and the precipitation of serum proteins was enhanced by ice-cooling for 2 min. After centrifugation, supernatant and precipitate were measured for radioactivity and the supernatant was analyzed by analytical radio-HPLC. These experiments were performed thrice.

**Cell culture**. All cell lines were grown in suitable culture medium at 37 °C in a humidified CO_2_ (5%) atmosphere. The human breast cancer cell lines T-47D, MDA-MB-231 and MCF-7 were grown in Dulbecco’s Modified Eagle Medium (DMEM), supplemented with 10% (*v*/*v*) fetal calf serum (FCS) and 1% (*v*/*v*) L-Glutamine. For a high expression of the NPY(Y_1_) receptor on T-47D cells, the medium for this cell line was further supplemented with 0.15% (*w*/*v*) β-estradiol. The BT-474 and PC-3 cell lines were grown in RPMI-1640 medium, also supplemented with 10% (*v*/*v*) fetal calf serum (FCS) and 1% (*v*/*v*) L-Glutamine.

**Internalization studies**. Cells (T-47D, MDA-MB-231, MCF-7, BT-474 and PC-3, 1.5 × 10^6^ cells per well) were seeded into 6-well plates and incubated overnight at 37 °C in a humidified CO_2_ (5%) atmosphere. The next day, the medium was removed and the cells were washed twice with the respective medium without supplements (ice-cold, 1 mL) and incubated with 3.7–4.0 kBq (0.37–4.0 pmol) of the respective ^68^Ga-radiolabeled ligand [^68^Ga]**22**−[^68^Ga]**26**, [^68^Ga]**27** or [^68^Ga]**28** (in 1.5 mL medium, containing 0.5% (*w*/*v*) BSA) for defined time-points of 1, 2, 3 or 4 h at 37 °C in a humidified CO_2_ (5%) atmosphere. A 1000-fold excess of the respective peptide (**3** or **4**) was used for blocking to determine the non-specific cell uptake. At each time point, the medium was removed and the cells were washed twice with the respective medium without supplements (ice-cold, 1 mL). Cells were treated twice with 1 mL glycine buffer (ice-cold, 50 mM glycine, 100 mM NaCl, pH 2.8) for 5 min at room temperature, followed by 2 mL NaOH solution (1 M) for 10 min at 37 °C. The supernatants were collected and the radioactivity measured in a gamma counter. The internalized and surface bound activity was expressed as percentage of measured to total added activity. Each data point was generated thrice in triplicates.

These internalization studies were performed accordingly on T-47D, MCF-7, BT-474 and PC-3 (negative control) cells with ^125^I-PYY (PerkinElmer, molar activity 81.4 GBq/µmol, 0.3 kBq, 0.0032 pmol). The cells were incubated for 1 h with the radioligand and additional blocking experiments were performed using a 1000-fold excess of **4**, **28** and **24**.

**In vivo experiments**. All animal experiments were performed in compliance with the German animal protection laws and protocols of the local committee (Regierungspräsidium Karlsruhe, approval number: 35-9185.81/G-206/15). 20, six week old female immunodeficient Fox Chase SCID (CB17/Icr-Prkdc^scid^/IcrIcoCrl) mice with an average weight of 20 g were subcutaneously implanted with 17β-estradiol pellets (0.36 mg/60 days). 4 days later, the tumors were induced by subcutaneous inoculation of 5 × 10^6^ T-47D cells into the left flank of the approval number s. After induction, the tumors were allowed to grow for 8–10 weeks and reached a diameter of about 0.5 cm. For imaging, the animals were anaesthetized with isoflurane and injected with 5.5–8.0 MBq of the respective radioligand ([^68^Ga]**24**, [^68^Ga]**24a** or [^68^Ga]**24b**) into the lateral tail vein. Dynamic PET images were acquired over 90 min and CT images were obtained within further 30 min. After the end of the diagnostic scan, the animals were sacrificed, the organs were collected and measured in a gamma-counter.

The dynamic PET images were reconstructed using the Albira Suite Reconstructor (Bruker) with an iterative dynamic reconstruction with 12 iterations using an 2D-Maximum-Likelihood Expectation-Maximization (MLEM) algorithm and a cubic image voxel size of 0.5 mm after scatter and decay correction. Data were divided into time frames from 1 to 10 min (10 × 1 min, 10 × 2 min, 6 × 5 min and 3 × 10 min) for the assessment of temporal changes in regional tracer accumulation. The CT images were obtained at 45 kVp, with currents of 0.4 mA (high dose, good resolution). Acquisitions of 400 projections were taken and a 250 μm isotropic voxel size image was reconstructed via filtered back projection. The reconstructed PET data were manually fused with the CT images using PMOD 3.6.1.1. and analyzed. Volumes of interest (VOIs) were defined for the quantification of tracer accumulation in heart, liver, kidneys, tumor, and muscle. The results for each VOI were calculated as SUV (kBq/cm^3^) averaged for each time frame.

## 4. Conclusions

We were able to show that is chemically and radiochemically feasible to synthesize radiolabeled heterobivalent peptides consisting of a GRPR- and a NPY(Y_1_)R-affine peptide on symmetrically branched scaffolds, resulting in bispecific heterobivalent peptidic PET radiotracers. The compounds demonstrated high stabilities in human serum, hydrophilicities comparable to the monomeric lead peptides and high GRPR-mediated tumor cell uptakes in vitro.

The performed in vivo imaging and ex vivo biodistribution studies indicated a contribution of both peptides of the evaluated HBPL to overall in vivo tumor uptake, showing the feasibility of the general concept to develop GRPR- and NPY(Y_1_)R-bispecific PET radiotracers with regard to an improved and more sensitive tumor visualization of human breast cancer.

Nevertheless, the results also show that further work is required to obtain GRPR- and NPY(Y_1_)R-bispecific imaging agents being useful for clinical application due to the high kidney and liver accumulation of the agents developed so far.

## Supplementary Materials

Supporting material for this article, comprising the syntheses of compounds **7**–**11** and **12**–**16**, the results of the log*_D_* determinations, radio-HPLC chromatograms and serum stability data, results of in vitro cell uptake studies and ex vivo biodistribution data is available online at http://www.mdpi.com/1424-8247/11/3/65/s1.
