# Supplementary material for: Design, Synthesis, In Vitro, and Initial In Vivo Evaluation of Heterobivalent Peptidic Ligands Targeting Both NPY(Y1)- and GRP-Receptors—An Improvement for Breast Cancer Imaging?
Source: Pharmaceuticals (Basel). 2018 Jul 4;11(3):65. doi: 10.3390/ph11030065 (PMC6161111; doi:10.3390/ph11030065)
Supplement: Supplementary file 1 [file pharmaceuticals-11-00065-s001.pdf]

pharmaceuticals

SUPPLEMENTARY INFORMATION FOR MANUSCRIPT

Design, Synthesis, *In vitro* and Initial *In Vivo* Evaluation of Heterobivalent Peptidic Ligands Targeting Both NPY(Y_1)- and GRP-Receptors − An Improvement for Breast Cancer Imaging?

Alicia Vall-Sagarra, Shanna Litau, Clemens Decristoforo, Björn Wängler, Ralf Schirrmacher, Gert Fricker, Carmen Wängler*

Table of Contents

Description of syntheses of NODA-GA-*bis*-amines (**7** − **11**) and NODA-GA-*bis*-aldehydes (**12** − **16**)	page 2
Typical analytical radio-HPLC chromatograms of [^{68}Ga]**22** – [^{68}Ga]**26**, [^{68}Ga]**27** and [^{68}Ga]**28** directly after ^{68}Ga-radiolabeling and after 90 minutes incubation with human serum at 37°C (Fig. S1)	page 4
Results of the log$_D$ determinations for the HBPLs and monomeric reference substances **[^{68}Ga]22** − **[^{68}Ga]26**, **[^{68}Ga]27** and **[^{68}Ga]28** (Fig. S2)	page 5
Results of *in vitro* cell uptake studies of **[^{68}Ga]22** (Fig. S3), **[^{68}Ga]23** (Fig. S4), **[^{68}Ga]25** (Fig. S5), **[^{68}Ga]26** (Fig. S6) and **[^{68}Ga]27** (Fig. S7) on T-47D cells, differentiated by overall uptake, internalization and surface binding	page 6
Results of *in vitro* cell uptake studies of **[^{68}Ga]27** and **[^{68}Ga]28** on BT-474 (Fig. S8), MCF-7 (Fig. S9) and MDA-MB-231 cells (Fig. S10)	page 8
Table S1: *Ex vivo* biodistribution data of [^{68}Ga]**24**, [^{68}Ga]**24a** and [^{68}Ga]**24b** in T-47D tumor-bearing mice at 130 min p.i.	page 10

General synthesis of the branched NODA-GA-*bis*-amines (7 − 11). The branched NODA-GA-*bis*-amines were synthesized by standard solid phase-based synthesis methods by coupling Fmoc-Lys(Mtt)-OH and Fmoc-PEG_4-OH to a low loading NovaPEG Rink amide resin. In the following, the side chain Mtt-protecting group of the lysine was removed using diluted TFA (TFA : DCM 1 : 99 (v/v)) within 2 h and NODA-GA-(*t*Bu)$_3$ was coupled in this position within 120 minutes using an excess of the synthon of 3 eq. together with 2.9 eq. PyBOP and 6 eq. DIPEA. Afterwards, *N*,*N*-bis(*N*'-Fmoc-3-aminopropyl)-glycine potassium hemisulfate was coupled under standard conditions, followed (where applicable) by Fmoc-PEG_2-OH, Fmoc-PEG_4-OH or one or two copies of Fmoc-ACMP (each applied in 8-fold excess together with 7.9 eq. HBTU and 8 eq. DIPEA). The resulting NODA-GA-*bis*-amines (**7** − **11**) were cleaved from the solid support using a mixture of TFA : TIS : H_2O of 90 : 5 : 5 (v/v) for 3h and purified by semipreparative HPLC after evaporation of the volatile materials. The products were isolated as colorless, hardening oils after lyophilization. Gradients used for HPLC purification and synthesis yields for each compound are given below.

7: gradient: 0–22.5% MeCN + 0.1% TFA in 6 min (R_t = 4.76 min), yield: 51%. MALDI-MS (m/z) using α-cyano-4-hydroxycinnamic acid as matrix substance for $[M+H^+]^+$ (calculated): 921.43 (921.55); $[M+Na^+]^+$ (calculated): 943.46 (943.54); $[M+K^+]^+$ (calculated): 959.43 (959.52). MALDI-MS (m/z) using 2,5-dihydroxybenzoic acid as matrix substance for $[M+H^+]^+$ (calculated): 921.24 (921.55); $[M+Na^+]^+$ (calculated): 943.17 (943.54); $[M+K^+]^+$ (calculated): 959.17 (959.52).

8: gradient: 0–25% MeCN + 0.1% TFA in 6 min (R_t = 4.76 min), yield: 57%. MALDI-MS (m/z) using α-cyano-4-hydroxycinnamic acid as matrix substance for $[M+H^+]^+$ (calculated): 1211.77 (1211.70); $[M+Na^+]^+$ (calculated): 1233.76 (1233.69); $[M+K^+]^+$ (calculated): 1249.71 (1249.67). MALDI-MS (m/z) using 2,5-dihydroxybenzoic acid as matrix substance for $[M+H^+]^+$ (calculated): 1211.56 (1211.70); $[M+Na^+]^+$ (calculated): 1233.59 (1233.69).

9: gradient: 0–25% MeCN + 0.1% TFA in 6 min (R_t = 5.64 min), yield: 59%. MALDI-MS (m/z) using α-cyano-4-hydroxycinnamic acid as matrix substance for $[M+H^+]^+$ (calculated): 1416.06 (1415.84); $[M+Na^+]^+$ (calculated): 1438.07 (1437.83); $[M+K^+]^+$ (calculated): 1454.01 (1453.80). MALDI-MS (m/z) using 2,5-dihydroxybenzoic acid as matrix substance for $[M+H^+]^+$ (calculated): 1415.71 (1415.84). MALDI-MS (m/z) using sinapic acid as matrix substance for $[M+H^+]^+$ (calculated): 1415.57 (1415.84); $[M+Na^+]^+$ (calculated): 1437.73 (1437.83); $[M+K^+]^+$ (calculated): 1453.54 (1453.80).

10: gradient: 0–20% MeCN + 0.1% TFA in 5 min (R_t = 4.50 min), yield: 49%. MALDI-MS (m/z) using α-cyano-4-hydroxycinnamic acid as matrix substance for $[M+H^+]^+$ (calculated): 1202.28 (1201.74). MALDI-MS (m/z) using 2,5-dihydroxybenzoic acid as matrix substance for $[M+H^+]^+$ (calculated): 1201.73 (1201.74); $[M+Na^+]^+$ (calculated): 1223.69 (1223.73); $[M+K^+]^+$ (calculated): 1239.66 (1239.71). MALDI-MS (m/z) using sinapic acid as matrix substance for $[M+H^+]^+$ (calculated): 1201.71 (1201.74); $[M+Na^+]^+$ (calculated): 1223.66 (1223.73); $[M+K^+]^+$ (calculated): 1239.67 (1239.71).

11: gradient: 0–20% MeCN + 0.1% TFA in 5 min (R_t = 4.48min), yield: 53%. MALDI-MS (m/z) using α-cyano-4-hydroxycinnamic acid as matrix substance for $[M+H^+]^+$ (calculated): 1482.52 (1481.93). MALDI-MS (m/z) using 2,5-dihydroxybenzoic acid as matrix substance for $[M+H^+]^+$ (calculated): 1481.35 (1481.93); $[M+Na^+]^+$ (calculated): 1503.42 (1503.92); $[M+K^+]^+$ (calculated): 1519.34 (1519.90).

General synthesis of the branched NODA-GA-*bis*-aldehydes (12 − 16). To a solution of the respective branched NODA-GA-*bis*-amines (**7 − 11)** in H_2O + 0.1% TFA (500 µL) was added a solution of SFB (2.5 − 5 eq.) in MeCN + 0.1% TFA (400 µL). The pH of the solutions was adjusted to 6.5 - 7.0 by addition of phosphate buffer (0.5M, pH 7.2, ~250 µL), precipitated SFB was redissolved by addition of MeCN (100 − 250 µL) and the reaction progress was monitored by analytical HPLC. After 1 to 4.5h, the reactions were complete and the products were purified by semipreparative HPLC. The products were isolated as white solids after lyophilization. Gradients used for HPLC purification and synthesis yields for each compound are given below.

12: gradient: 20–30% MeCN + 0.1% TFA in 5 min (R_t = 3.02 min), yield: 51%. MALDI-MS (m/z) using α-cyano-4-hydroxycinnamic acid as matrix substance for $[M+H^+]^+$ (calculated): 1185.86 (1185.60); $[M+Na^+]^+$ (calculated): 1207.85 (1207.59); $[M+K^+]^+$ (calculated): 1223.79 (1223.56). MALDI-MS (m/z) using 2,5-dihydroxybenzoic acid as matrix substance for $[M+H^+]^+$ (calculated): 1185.55 (1185.60); $[M+Na^+]^+$ (calculated): 1207.68 (1207.59); $[M+K^+]^+$ (calculated): 1223.78 (1223.56).

13: gradient: 20–25% MeCN + 0.1% TFA in 5 min (R_t = 3.65 min), yield: 39%. MALDI-MS (m/z) using α-cyano-4-hydroxycinnamic acid as matrix substance for $[M+H^+]^+$ (calculated): 1475.32 (1475.74); $[M+Na^+]^+$ (calculated): 1497.38 (1497.73); $[M+K^+]^+$ (calculated): 1513.36 (1513.71). MALDI-MS (m/z) using 2,5-dihydroxybenzoic acid as matrix substance for $[M+H^+]^+$ (calculated): 1475.32 (1475.74); $[M+Na^+]^+$ (calculated): 1497.32 (1497.73); $[M+K^+]^+$ (calculated): 1513.34 (1513.71). MALDI-MS (m/z) using sinapic acid as matrix substance for $[M+H^+]^+$ (calculated): 1475.77 (1475.74); $[M+Na^+]^+$ (calculated): 1497.92 (1497.73); $[M+K^+]^+$ (calculated): 1513.76 (1513.71).

14: gradient: 20–30% MeCN + 0.1% TFA in 5 min (R_t = 4.65 min), yield: 42%. MALDI-MS (m/z) using α-cyano-4-hydroxycinnamic acid as matrix substance for $[M+H^+]^+$ (calculated): 1680.17 (1679.88). MALDI-MS (m/z) using 2,5-dihydroxybenzoic acid as matrix substance for $[M+H^+]^+$ (calculated): 1679.31 (1679.88); $[M+Na^+]^+$ (calculated): 1701.43 (1701.87); $[M+K^+]^+$ (calculated): 1717.42 (1717.84).

15: gradient: 5–30% MeCN + 0.1% TFA in 6 min (R_t = 5.48 min), yield: 66%. MALDI-MS (m/z) using α-cyano-4-hydroxycinnamic acid as matrix substance for $[M+H^+]^+$ (calculated): 1465.94 (1465.79); $[M+Na^+]^+$ (calculated): 1487.96 (1487.78); $[M+K^+]^+$ (calculated): 1503.96 (1503.75). MALDI-MS (m/z) using 2,5-dihydroxybenzoic acid as matrix substance for $[M+H^+]^+$ (calculated): 1465.93 (1465.79). MALDI-MS (m/z) using sinapic acid as matrix substance for $[M+H^+]^+$ (calculated): 1465.35 (1465.79); $[M+Na^+]^+$ (calculated): 1487.45 (1487.78); $[M+K^+]^+$ (calculated): 1503.32 (1503.75).

16: gradient: 5–30% MeCN + 0.1% TFA in 7 min (R_t = 5.06 min), yield: 57%. MALDI-MS (m/z) using α-cyano-4-hydroxycinnamic acid as matrix substance for $[M+H^+]^+$ (calculated): 1745.95 (1745.98); $[M+Na^+]^+$ (calculated): 1767.94 (1767.97). MALDI-MS (m/z) using 2,5-dihydroxybenzoic acid as matrix substance for $[M+H^+]^+$ (calculated): 1745.79 (1745.98); $[M+Na^+]^+$ (calculated): 1767.80 (1767.97); $[M+K^+]^+$ (calculated): 1783.69 (1783.94).

Typical analytical radio-HPLC chromatograms of [^{68}Ga]22 − [^{68}Ga]26, [^{68}Ga]27 and [^{68}Ga]28 directly after ^{68}Ga-radiolabeling and after 90 minutes incubation with human serum at 37°C.

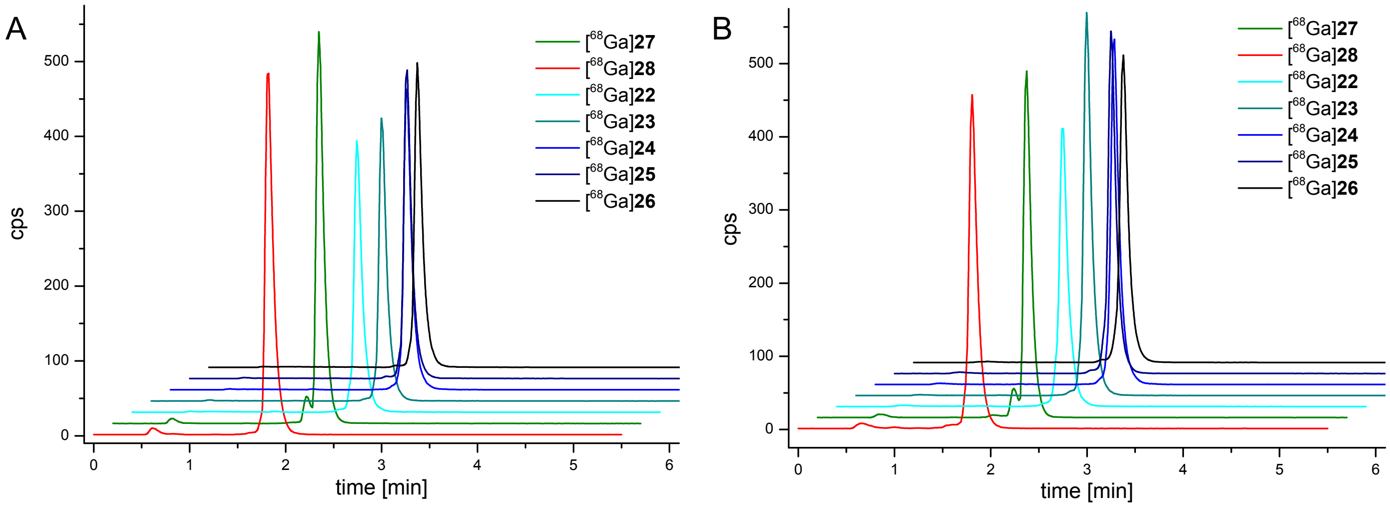

Fig. S1: Typical analytical radio-HPLC chromatograms of [^{68}Ga]**22** − [^{68}Ga]**26**, [^{68}Ga]**27** and [^{68}Ga]**28** directly after ^{68}Ga-radiolabeling (**A**) and after 90 minutes incubation with human serum at 37°C (**B**).

Results of the log$_D$ determinations for the HBPLs and monomeric reference substances [^{68}Ga]22 – [^{68}Ga]26, [^{68}Ga]27 and [^{68}Ga]28

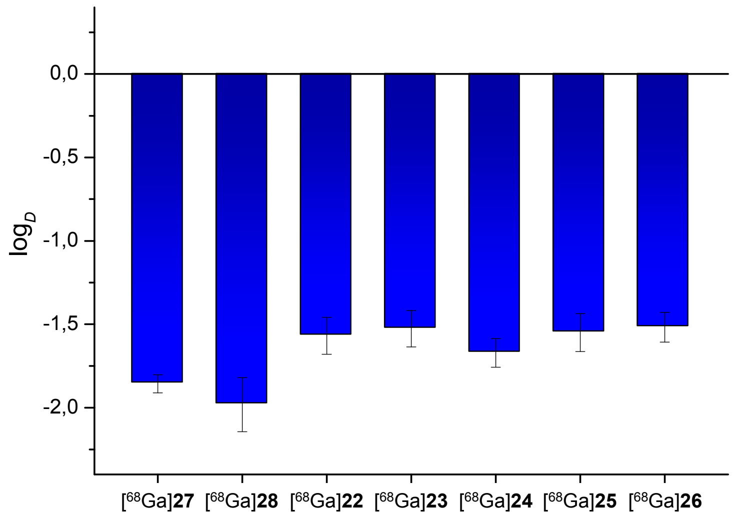

Fig. S2: Depiction of the results of the log$_D$ determinations for the HBPLs **[^{68}Ga]22** – **[^{68}Ga]26** in comparison to the monomeric reference peptides **[^{68}Ga]27** and **[^{68}Ga]28**.

Results of *in vitro* cell uptake studies of [^{68}Ga]22 (Fig. S3), [^{68}Ga]23 (Fig. S4), [^{68}Ga]25 (Fig. S5), [^{68}Ga]26 (Fig. S6) and [^{68}Ga]27 (Fig. S7) on T-47D cells, differentiated by overall uptake, internalization and surface binding.

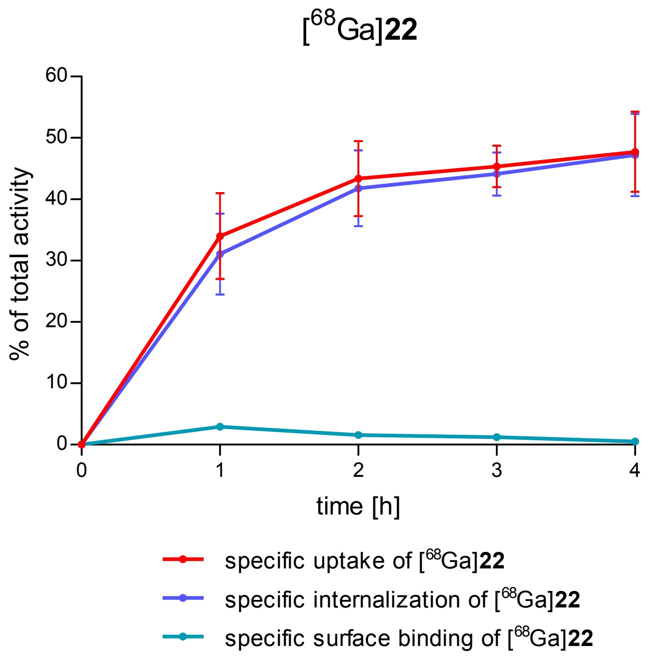

Fig. S3:
Specific cell uptake of **[^{68}Ga]22** into T-47D cells over 4h of incubation.

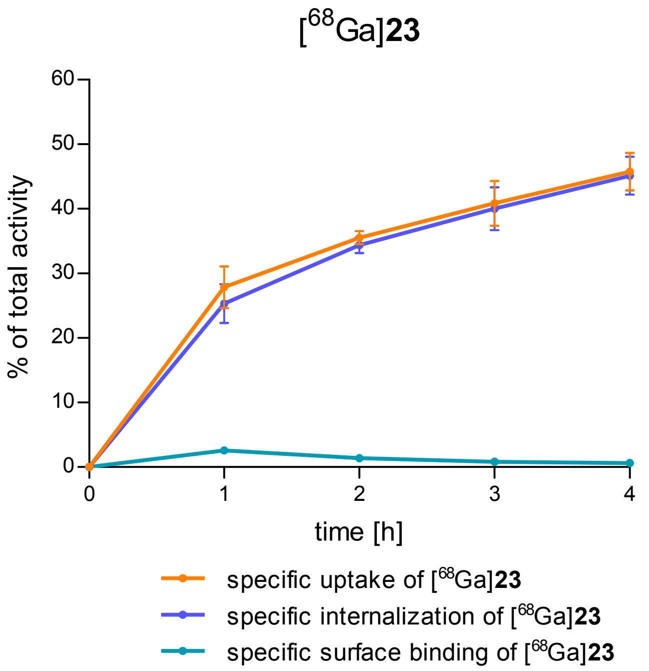

Fig. S4:
Specific cell uptake of **[^{68}Ga]23** into T-47D cells over 4h of incubation.

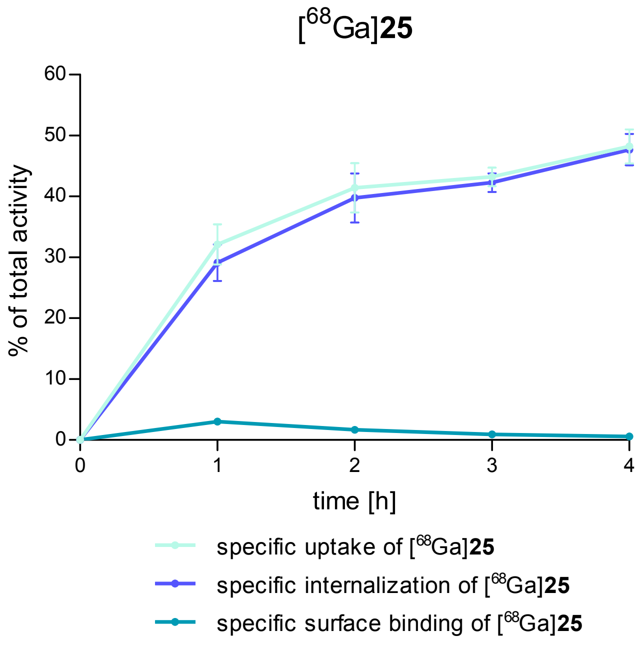

Fig. S5:
Specific cell uptake of **[^{68}Ga]25** into T-47D cells over 4h of incubation.

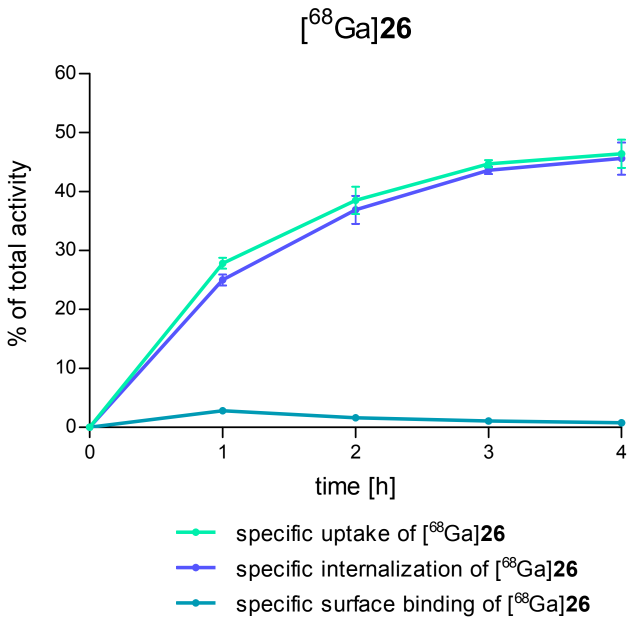

Fig. S6:
Specific cell uptake of **[^{68}Ga]26** into T-47D cells over 4h of incubation.

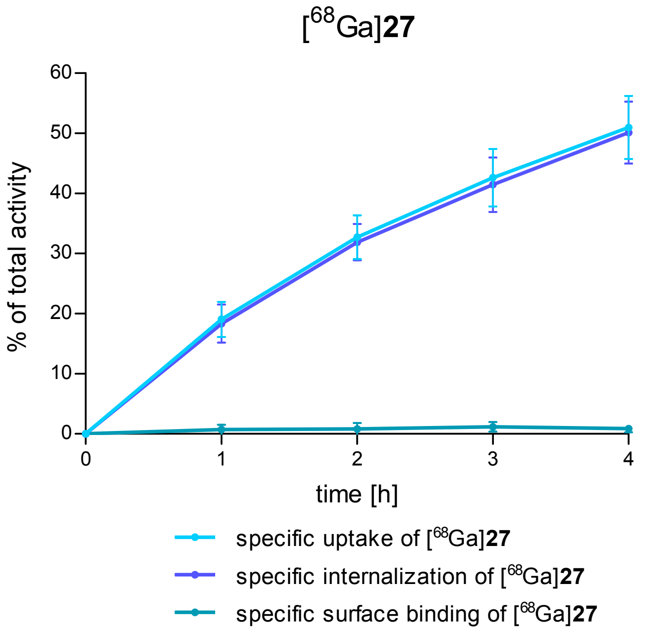

Fig. S7:
Specific cell uptake of **[^{68}Ga]27** into T-47D cells over 4h of incubation.

Results of *in vitro* cell uptake studies of [^{68}Ga]27 and [^{68}Ga]28 on MDA-MB-231, MCF-7 und BT-474 cells.

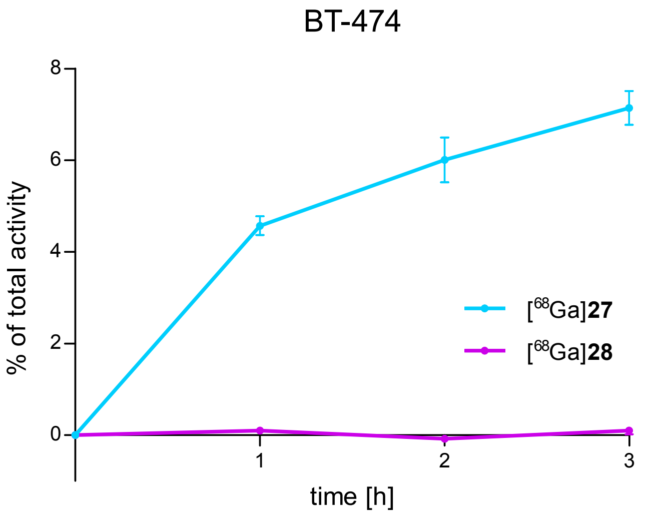

Fig. S8:
Specific cell uptake of **[^{68}Ga]27** and **[^{68}Ga]28** into BT-474 cells over 4h of incubation.

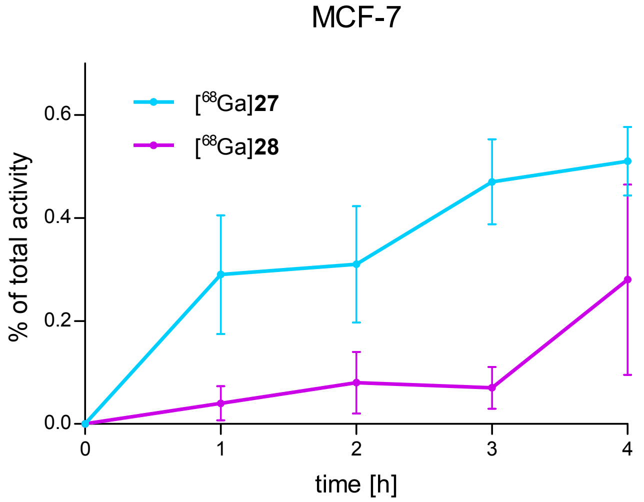

Fig. S9:
Specific cell uptake of **[^{68}Ga]27** and **[^{68}Ga]28** into MCF-7 cells over 4h of incubation.

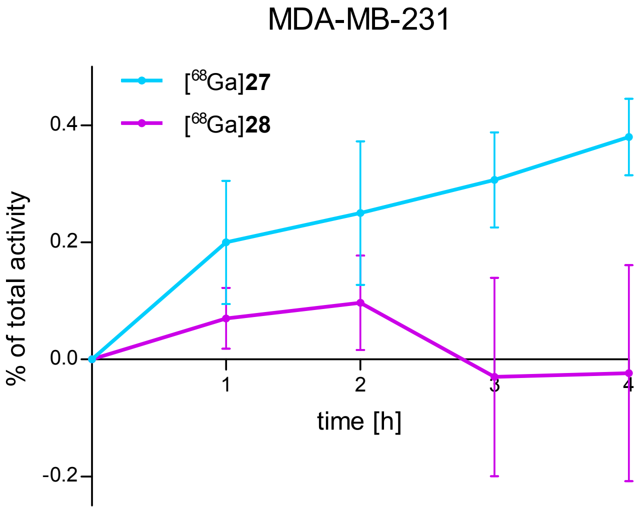

Fig. S10:
Specific cell uptake of **[^{68}Ga]27** and **[^{68}Ga]28** into MDA-MB-231 cells over 4h of incubation.

Table S1: *Ex vivo* biodistribution data (ID/g in %) of [^{68}Ga]24, [^{68}Ga]24a and [^{68}Ga]24b in T-47D tumor-bearing mice at 130 min p.i.

organ	[^{68}Ga]**24**	[^{68}Ga]**24a**	[^{68}Ga]**24b**
tumor	3.07 ± 0.33	0.21 ± 0.15	1.84 ± 0.32
blood	1.13 ± 0.77	0.39 ± 0.37	2.02 ± 1.34
heart	0.52 ± 0.38	0.18 ± 0.14	0.85 ± 0.52
lung	0.62 ± 0.17	0.28 ± 0.04	0.88 ± 0.07
stomach	1.47 ± 0.44	0.39 ± 0.41	1.35 ± 0.43
liver	10.02 ± 1.54	5.54 ± 0.31	16.75 ± 0.85
small intestines	1.49 ± 0.36	0.43 ± 0.37	1.53 ± 0.51
large intestines	1.80 ± 0.64	0.24 ± 0.19	1.71 ± 0.69
pancreas	10.54 ± 3.13	0.15 ± 0.08	8.30 ± 5.33
spleen	1.32 ± 0.33	0.49 ± 0.14	2.12 ± 0.49
kidneys	34.79 ± 11.03	29.72 ± 10.33	43.84 ± 25.05
adrenal glands	3.57 ± 2.18	0.74 ± 0.40	2.91 ± 1.13
muscle	0.26 ± 0.18	0.26 ± 0.40	0.48 ± 0.40
bone	0.26 ± 0.14	0.13 ± 0.08	0.43 ± 0.23
brain	0.11 ± 0.14	0.03 ± 0.02	0.07 ± 0.04
tail	1.30 ± 1.21	0.46 ± 0.41	1.64 ± 0.94
T / B	2.72 ± 0.43	0.50 ± 0.47	0.91 ± 0.24
T / M	11.81 ± 1.83	1.07 ± 1.14	3.83 ± 0.80
